# Rad51 recruitment and exclusion of non-homologous end joining during homologous recombination at a Tus/*Ter* mammalian replication fork barrier

**DOI:** 10.1371/journal.pgen.1007486

**Published:** 2018-07-19

**Authors:** Nicholas A. Willis, Arvind Panday, Erin E. Duffey, Ralph Scully

**Affiliations:** Department of Medicine, Division of Hematology-Oncology and Cancer Research Institute, Beth Israel Deaconess Medical Center and Harvard Medical School, Boston, Massachusetts, United States of America; University of Iowa, UNITED STATES

## Abstract

Classical non-homologous end joining (C-NHEJ) and homologous recombination (HR) compete to repair mammalian chromosomal double strand breaks (DSBs). However, C-NHEJ has no impact on HR induced by DNA nicking enzymes. In this case, the replication fork is thought to convert the DNA nick into a one-ended DSB, which lacks a readily available partner for C-NHEJ. Whether C-NHEJ competes with HR at a non-enzymatic mammalian replication fork barrier (RFB) remains unknown. We previously showed that conservative “short tract” gene conversion (STGC) induced by a chromosomal Tus/*Ter* RFB is a product of bidirectional replication fork stalling. This finding raises the possibility that Tus/*Ter*-induced STGC proceeds *via* a two-ended DSB intermediate. If so, Tus/*Ter*-induced STGC might be subject to competition by C-NHEJ. However, in contrast to the DSB response, where genetic ablation of C-NHEJ stimulates HR, we report here that Tus/*Ter*-induced HR is unaffected by deletion of either of two C-NHEJ genes, *Xrcc4* or *Ku70*. These results show that Tus/*Ter*-induced HR does not entail the formation of a two-ended DSB to which C-NHEJ has competitive access. We found no evidence that the alternative end-joining factor, DNA polymerase θ, competes with Tus/*Ter*-induced HR. We used chromatin-immunoprecipitation to compare Rad51 recruitment to a Tus/*Ter* RFB and to a neighboring site-specific DSB. Rad51 accumulation at Tus/*Ter* was more intense and more sustained than at a DSB. In contrast to the DSB response, Rad51 accumulation at Tus/*Ter* was restricted to within a few hundred base pairs of the RFB. Taken together, these findings suggest that the major DNA structures that bind Rad51 at a Tus/*Ter* RFB are not conventional DSBs. We propose that Rad51 acts as an “early responder” at stalled forks, binding single stranded daughter strand gaps on the arrested lagging strand, and that Rad51-mediated fork remodeling generates HR intermediates that are incapable of Ku binding and therefore invisible to the C-NHEJ machinery.

## Author summary

Genomic instability is a significant contributor to human disease, ranging from hereditary developmental disorders to cancer predisposition. Two major triggers to genomic instability are chromosomal double strand breaks (DSBs) and the stalling of replication forks during the DNA synthesis (S phase) of the cell cycle. The “rules” that govern mammalian DSB repair are increasingly well understood, and it is recognized that the two major DSB repair pathways—classical non-homologous end joining (C-NHEJ) and homologous recombination (HR)—compete to repair a mammalian DSB. In contrast, we do not yet have equivalent insight into the regulation of repair at sites of mammalian replication fork stalling. Here, we explore the relationship between C-NHEJ and HR at a defined chromosomal replication fork barrier in mammalian cells. We show that, in contrast to DSB repair, repair at stalled forks does not entail competition between C-NHEJ and HR. We find that Rad51, a key mediator of HR, accumulates in an intense and highly localized fashion at the stalled fork. Based upon these findings, we propose a model of HR initiation at the stalled fork in which a Rad51-mediated fork remodeling step prevents access of C-NHEJ to the stalled fork.

## Introduction

The stalling of replication forks at sites of abnormal DNA structure, following collisions with transcription complexes or due to nucleotide pool depletion—collectively termed “replication stress”—is a significant contributor to genomic instability. Inherited mutations in genes that regulate the replication stress response cause a number of human diseases, ranging from developmental disorders to highly penetrant cancer predisposition syndromes [1–5]. Replication stress is thought to be a near-universal phenomenon in tumorigenesis and some of the molecules that act upon the stalled fork are considered promising targets for cancer therapy [6]. Replication fork stalling provokes a diverse set of cellular responses, including: stabilization of the stalled replisome; regulated replisome disassembly (“fork collapse”); protection of the fork from deleterious nucleolytic processing; remodeling of DNA structure at the stalled fork; and engagement of repair or “replication restart” [5, 7–15]. The S phase checkpoint and the homologous recombination (HR) systems are intimately involved in coordinating these responses, collaborating to suppress deleterious genome rearrangements at the stalled fork [2, 16–20]. However, the mechanisms governing this coordination remain poorly understood in mammalian cells.

DNA structure at the stalled fork is remodeled by topological stresses on the chromosome at the site of stalling and by the direct action of remodeling enzymes [5, 12, 21]. The fork can be reversed to form a Holliday junction, generating a solitary DNA end which is extensively single stranded due to accompanying nascent lagging strand resection [20, 22, 23]. Other forms of template switching can also occur in the vicinity of the stall site [18, 24, 25]. Endonuclease-mediated fork breakage—either scheduled or unscheduled—can generate double strand breaks (DSBs), which might be either one-ended or two-ended [5, 20]. The DNA structures generated by fork remodeling presumably limit the repair pathways that can be engaged. Two-ended DSBs can potentially be repaired by end joining mechanisms as well as by recombination [26, 27]. In contrast, a one-ended DSB or a solitary DNA end lacks a readily available ligation partner for end joining, and may preferentially engage break-induced replication [28, 29]. Consistent with this, HR induced by a two-ended chromosomal DSB is subject to competition by classical non-homologous end joining (C-NHEJ), whereas HR induced by a nicking enzyme (“nickase”)—in which the replication fork converts the nick into a one-ended DSB—is unaffected by deletion of C-NHEJ genes [30–32]. Thus, in mammalian cells, the susceptibility of HR to competition by C-NHEJ in a particular cellular context is a useful “probe” with which to analyze the DNA structural intermediates of HR. Since the stalled fork response entails the formation of diverse DNA structures and is not restricted to two-ended DSBs, repair pathway “choice” at a stalled fork may differ from that at a defined two-ended DSB.

Study of replication-coupled repair of a covalent DNA inter-strand crosslink (ICL) in *Xenopus laevis* egg extracts has revealed some of the fundamental steps of stalled fork processing and repair [2, 20]. The Fanconi anemia (FA)/BRCA pathway plays a key role in detecting and processing forks bidirectionally arrested at the ICL [33–35]. The FANCD2/FANCI heterodimer orchestrates dual incisions of one of the sister chromatids on either side of the ICL. Importantly, efficient incision of the bidirectionally arrested forks is suppressed until the two opposing forks have each stalled at the ICL [36]. The resulting two-ended DSB is repaired by HR-mediated sister chromatid recombination, in which the *BRCA* gene products play canonical roles in promoting Rad51 loading and strand exchange functions [37–41]. HR repair of such a two-ended DSB intermediate could, in principle, be subject to competition by C-NHEJ or other end joining systems. However, recent evidence of fork reversal during ICL repair suggests that at least one of the two DSB ends is extensively single stranded [23].

Competition between HR and C-NHEJ is not a major feature of DSB repair in yeast, since C-NHEJ is a relatively low-flux pathway. Additionally, the Fanconi anemia pathway in yeast is limited to evolutionarily conserved homologs of FANCM [42, 43], suggesting that the innovation of FANCD2/FANCI-coordinated incision of bidirectionally arrested forks occurred relatively recently in evolution. Thus, although certain “core” elements of DSB repair and stalled fork metabolism are conserved between yeast and vertebrates, there are likely significant inter-species differences that remain to be fully defined. Studies in yeast, using non-enzymatic, locus-specific replication fork barriers (RFBs), show that stalled fork HR can mediate both conservative and deleterious repair, the latter including gross chromosomal rearrangements and more localized copy number changes at the site of stalling [14, 18, 19, 24, 44–48]. In contrast to the above-noted *X*. *laevis* ICL repair model, HR at an *RTS1* RFB in *Schizosaccharomyces pombe* is not accompanied by evidence of DSB formation [19, 24]. Processing of the stalled fork in *S*. *pombe* may also trigger an aberrant form of “replication restart”, a *rad22*^Rad52^-dependent process in which the restarted fork is prone to collapse [45]. This aberrant fork restart mechanism is reminiscent of break-induced replication (BIR) in *Saccharomyces cerevisiae*, which is characteristically unstable and mutation-prone [49, 50]. Indeed, current models of aberrant replication restart in *S*. *pombe* invoke a migrating bubble mechanism equivalent to the mechanism of BIR in *S*. *cerevisiae* [49]. Rad52-dependent pathways have also been implicated in stalled fork repair in mammalian cells [51–53].

To facilitate analysis of mammalian stalled fork metabolism and repair, we adapted the *Escherichia coli* Tus/*Ter* RFB for use in mammalian cells [54–58]. A chromosomally integrated array of six 23 bp *Ter* sites mediates Tus-dependent, locus-specific replication fork stalling and HR on a mammalian chromosome, enabling direct quantitation of the repair products of mammalian replication fork stalling. We showed that conservative “short tract” gene conversion (STGC) at Tus/*Ter* is positively regulated by BRCA1, BRCA2, Rad51 and the Fanconi anemia pathway—consistent with the idea that STGC represents a physiological HR response to fork stalling [56, 58]. In contrast, “long tract” gene conversion (LTGC)—an error-prone HR outcome in which a replicative mechanism copies several kilobases from the partner sister chromatid—is suppressed by BRCA1 and appears to be Rad51-independent. We recently identified a novel product of stalled fork repair in primary mouse cells lacking the hereditary breast/ovarian cancer predisposition gene, *Brca1*: the formation of small (2–6 kb) non-homologous or microhomology-mediated tandem duplications (TDs) [58]. Tus/*Ter*-induced TDs in *Brca1* mutant cells are mediated by a replication restart-bypass mechanism, which is completed by *Xrcc4*-dependent C-NHEJ. This finding, together with previous observations, suggests that C-NHEJ can access DNA ends positioned close to the site of fork stalling [59, 60]. Notably, Tus/*Ter*-induced STGC is a product of bidirectional replication fork stalling [56]. By analogy with the processing of forks bidirectionally arrested at an ICL in *X*. *laevis*, Tus/*Ter*-induced STGC might entail the formation of a two-ended DSB intermediate and might therefore be subject to competition by C-NHEJ. To test this hypothesis, we have analyzed the impact of deletion of the C-NHEJ genes *Xrcc4* and *Ku70* on Tus/*Ter*-induced HR.

## Results

### Impact of *Xrcc4* deletion on Tus/*Ter*-induced HR

**Fig 1:**
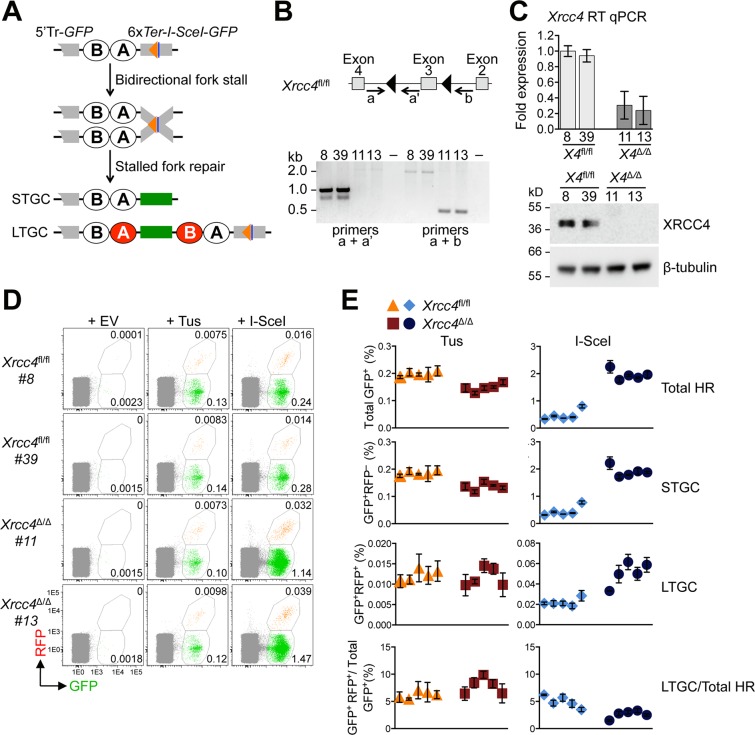
Impact of *Xrcc4* deletion on Tus/*Ter*-induced and I-SceI-induced HR. **A**, Schematic of 6x*Ter*-HR reporter and HR repair products of Tus-*Ter*-induced fork stalling. Green box: wt*GFP*. Grey boxes: mutant *GFP*. Open ovals A and B: 5’ and 3’ artificial *RFP* exons. 5’Tr-*GFP*: 5’-truncated *GFP*. Orange triangle: 6x*Ter* element array. Navy blue line: I-SceI endonuclease cut site. STGC, LTGC: short tract and long tract gene conversion HR repair outcomes. LTGC generates wt*RFP* through RNA splicing (red filled ovals). **B**, *Xrcc4* gene structure in *Xrcc4*^fl/fl^ ES cells. *Xrcc4*^Δ*/*Δ^ allele lacks exon 3. Black triangles: *loxP* sites. Grey boxes: *Xrcc4* Exons 2–4. Location and direction of Exon3 genotyping primers a, a’, and b as indicated by arrows. Gel: PCR products for *Xrcc4*^fl/fl^ ES clones 8 and 39, and *Xrcc4*^Δ*/*Δ^ clones 11 and 13. **C**, RT qPCR analysis of *Xrcc4* expression in *Xrcc4*^fl/fl^ or *Xrcc4*^Δ*/*Δ^ clones. *Xrcc4* expression normalized to *GAPDH* and displayed as fold difference from *Xrcc4*^fl/fl^ clone 8 of the same experiment (x = -2^ΔΔCt^, with ΔΔCt = [Ct_Xrcc4_-Ct_Gapdh_]-[Ct_*Xrcc4*_-Ct_*GAPDH*_]). Error-bars represent standard deviation of the ΔCt value (SDEV = √[SDEV_*Xrcc4*_^2^ + SDEV_*GAPDH*_^2^]). Xrcc4 abundance by Western blot in *Xrcc4*^fl/fl^ clones 8 and 39, and *Xrcc4*^Δ*/*Δ^ clones 11 and 13 cell protein extracts. **D**, Representative primary FACS data for two *Xrcc4*^fl/fl^ and two *Xrcc4*^Δ/Δ^ 6x*Ter*-HR reporter clones, as indicated, transfected with empty, 3xMyc-NLS Tus or 3xMyc-NLS I-SceI expression vectors. FACS plots produced from pooled data of duplicate samples from three independent experiments. Numbers represent percentages. **E**, Frequencies of Tus/*Ter*-induced and I-SceI-induced repair in five independently derived *Xrcc4*^fl/fl^ (orange triangles, red squares) or *Xrcc4*^Δ/Δ^ (blue diamonds, navy blue circles) 6x*Ter*-HR reporter clones transiently transfected with empty, Tus or I-SceI expression vectors. Each dot plot represents the mean of duplicate samples from three independent experiments (n = 3), values are corrected for transfection efficiency–see Materials and Methods. Error bars: standard error of the mean (s.e.m.). One-way ANOVA (Analysis of Variance) test comparing trend in HR between five *Xrcc4*^fl/fl^ and five *Xrcc4*^Δ/Δ^ clones: Tus-induced HR, total HR, p = 0.0017; STGC, p = 0.0015; LTGC, p = 0.7142; LTGC/(Total HR), p = 0.2636. I-SceI-induced HR, total HR, p<0.0001; STGC, p<0.0001; LTGC, p<0.0001; LTGC/(Total HR), p<0.0001. T-test comparing *Xrcc4*^fl/fl^ *vs*. *Xrcc4*^Δ/Δ^ clone pooled data, Tus-induced HR: total HR, p<0.0001; STGC, p<0.0001; LTGC, p = 0.6864; LTGC/(Total HR), p = 0.0332; I-SceI-induced HR, total HR, p<0.0001; STGC, p<0.0001; LTGC, p<0.0001; LTGC/(Total HR), p<0.0001.

To determine whether C-NHEJ interacts with HR at Tus/*Ter*-stalled replication forks, we targeted a 6x*Ter*-HR reporter as a single copy to the *ROSA26* locus of mouse embryonic stem (mES) cells carrying biallelic conditional alleles of the C-NHEJ gene *Xrcc4* (here termed “*Xrcc4*^fl/fl^”), as described in Materials and Methods [56, 61]. The 6x*Ter*-HR reporter contains an I-SceI target site adjacent to the 6x*Ter* array (**Fig 1A**). Thus, transfection of Tus enables analysis of HR in the stalled fork response, while transfection of I-SceI in parallel samples enables analysis of DSB-induced HR. The reporter also contains elements to distinguish short tract gene conversions (STGC) from long tract gene conversions (LTGC), the latter being rare HR products in wild type cells [62, 63]. Although HR by either STGC or LTGC converts the cell to GFP^+^, LTGC additionally converts the cell to RFP^+^, by replicative duplication of an *RFP* cassette within the reporter (**Fig 1A**) [56, 64].

We transduced a *ROSA26*-targeted *Xrcc4*^fl/fl^ 6x*Ter*-HR reporter clone with adenovirally-encoded Cre recombinase and screened for derivative clones that had either lost (*Xrcc4*^Δ/Δ^) or retained (*Xrcc4*^fl/fl^) *Xrcc4*. *Xrcc4* loss or retention was detected by PCR on genomic (g)DNA and was confirmed in a subset of clones by western blotting (**Fig 1B** and **1C**). We studied HR in five independent Cre-treated *Xrcc4*^fl/fl^ 6x*Ter*-HR reporter clones and five independent Cre-treated *Xrcc4*^Δ/Δ^ 6x*Ter*-HR reporter clones in response to either Tus or I-SceI—each transfected in parallel samples (see Materials and Methods). As expected, I-SceI-induced STGC and LTGC were elevated up to 4-fold in *Xrcc4*^Δ/Δ^ cells in comparison to *Xrcc4*^fl/fl^ cells (**Fig 1D** and **1E**) [30]. Interestingly, deletion of *Xrcc4* stimulated STGC more strongly than LTGC; as a result, the proportion of I-SceI-induced HR events that resolved as LTGC was reduced from ~5% in *Xrcc4*^fl/fl^ cells to ~2–3% in *Xrcc4*^Δ/Δ^ cells (**Fig 1E**). The impact of *Xrcc4* deletion on Tus/*Ter*-induced HR was quite different. Tus/*Ter*-induced STGC was marginally reduced in *Xrcc4*^Δ/Δ^ cells in comparison to *Xrcc4*^fl/fl^ cells, while Tus/*Ter*-induced LTGC was unaffected by deletion of *Xrcc4* (**Fig 1D** and **1E**). These results suggest that the interaction between HR and C-NHEJ at a chromosomal DSB is not recapitulated in the regulation of HR at a stalled replication fork.

**Fig 2:**
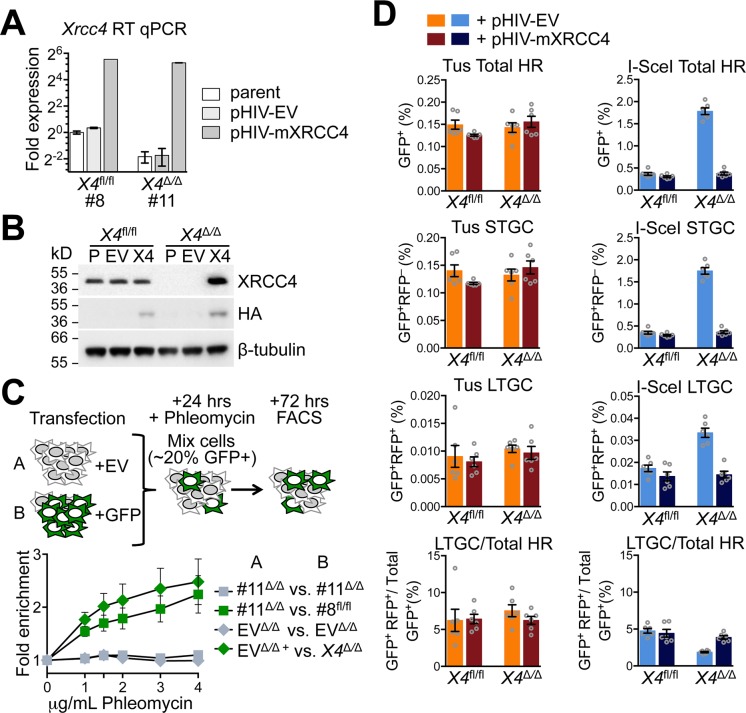
Stable re-expression of wt*Xrcc4* does not affect Tus/*Ter*-induced HR in *Xrcc4*^Δ/Δ^ cells. **A**, RT qPCR analysis of *Xrcc4* expression in stably transduced *Xrcc4*^fl/fl^ or *Xrcc4*^Δ*/*Δ^ clones. *Xrcc4* expression normalized to *GAPDH* and displayed as fold difference from *Xrcc4*^fl/fl^ parental reporter clone 8 of the same experiment (x = -2^ΔΔCt^, with ΔΔCt = [Ct_Xrcc4_-Ct_Gapdh_]-[Ct_*Xrcc4*_-Ct_*GAPDH*_]). Error-bars represent standard deviation of the ΔCt value (SDEV = √[SDEV_*Xrcc4*_^2^ + SDEV_*GAPDH*_^2^]). **B**, Xrcc4 protein abundance by Western blot in extracts of parental *Xrcc4*^fl*/*fl^ clone #8 and *Xrcc4*^Δ*/*Δ^ clone #11 and derivative cultures stably transduced with empty lentiviral vector (pHIV-NAT-hCD52, “EV”) or HA-tagged mouse Xrcc4 lentiviral expression vector (“X4”). **C**, Fold enrichment of cultures transiently expressing exogenous GFP. Results represent fold enrichment of cultures transiently co-transfected with pcDNA3beta and *GFP*-expression plasmid co-cultured cells transiently transfected with pcDNA3beta alone. Each plot represents the mean of triplicate samples from three independent experiments (n = 3), fold enrichment GFP+ cells normalized to 0 μg/mL phleomycin control. Error bars: s.e.m. **D**, Frequencies of Tus/*Ter*-induced and I-SceI-induced repair in *Xrcc4*^fl/fl^ clone #8 or *Xrcc4*^Δ/Δ^ clone #11 6x*Ter*-HR reporter cells lentivirally transduced with pHIV-NAT-hCD52-EV (empty vector control) or pHIV-NAT-hCD52-*mXrcc4* (expressing HA-tagged mouse Xrcc4 expression vector) with selection of transduced cells in 100 μg/ml NTC. Cells were transiently transfected with empty, 3xMyc-NLS Tus or 3xMyc-NLS I-SceI expression vectors. Each plot represents the mean of duplicate samples from six independent experiments (n = 6). Error bars: s.e.m. Tus-induced Total HR, t-test: flox8 +Xrcc4 *vs*. flox8 +EV p = 0.0662; del11 +Xrcc4 *vs*. del11 +EV p = 0.4509; del11 +EV *vs*. flox8 +EV p = 0.6719; del11 +Xrcc4 *vs*. flox8 +Xrcc4 p = 0.0588; del11 +Xrcc4 *vs*. flox8 +EV p = 0.5025. Tus-induced STGC, t-test: flox8 +Xrcc4 *vs*. flox8 +EV p = 0.0836; del11 +Xrcc4 *vs*. del11 +EV p = 0.4126; del11 +EV *vs*. flox8 +EV p = 0.6144; del11 +Xrcc4 *vs*. flox8 +Xrcc4 p = 0.0595; del11 +Xrcc4 *vs*. flox8 +EV p = 0.7215. Tus-induced LTGC, t-test: flox8 +Xrcc4 *vs*. flox8 +EV p = 0.6686; del11 +Xrcc4 *vs*. del11 +EV p = 0.5972; del11 +EV *vs*. flox8 +EV p = 0.5313; del11 +Xrcc4 *vs*. flox8 +Xrcc4 p = 0.3007; del11 +Xrcc4 *vs*. flox8 +EV p = 0.7870. Tus-induced LTGC/Total HR ratio, t-test: flox8 +Xrcc4 *vs*. flox8 +EV p = 0.9182; del11 +Xrcc4 *vs*. del11 +EV p = 0.2133; del11 +EV *vs*. flox8 +EV p = 0.4686; del11 +Xrcc4 *vs*. flox8 +Xrcc4 p = 0.8360; del11 +Xrcc4 *vs*. flox8 +EV p = 0.5771. I-SceI-induced Total HR, t-test: flox8 +Xrcc4 *vs*. flox8 +EV: p = 0.1292; del11 +Xrcc4 *vs*. del11 +EV p<0.0001; del11 +EV *vs*. flox8 +EV p<0.0001; del11 +Xrcc4 *vs*. flox8 +Xrcc4 p = 0.1030; del11 +Xrcc4 *vs*. flox8 +EV p = 0.8690. I-SceI-induced STGC, t-test: flox8 +Xrcc4 *vs*. flox8 +EV p = 0.1353; del11 +Xrcc4 *vs*. del11 +EV p<0.0001; del11 +EV *vs*. flox8 +EV p<0.0001; del11 +Xrcc4 *vs*. flox8 +Xrcc4 p = 0.0939; del11 +Xrcc4 *vs*. flox39 +EV p = 0.0081. I-SceI-induced LTGC, t-test: flox8 +Xrcc4 *vs*. flox8 +EV p = 0.1840; del11 +Xrcc4 *vs*. del11 +EV p<0.0001; del13 +EV vs. flox39 +EV p<0.0001; del11 +Xrcc4 *vs*. flox8 +Xrcc4 p = 0.7589; del11 +Xrcc4 *vs*. flox39 +EV p = 0.1347. I-SceI-induced LTGC/Total HR ratio, t-test: flox8 +Xrcc4 *vs*. flox8 +EV p = 0.5908; del11 +Xrcc4 *vs*. del11 +EV p = 0.0001; del11 +EV *vs*. flox8 +EV p = 0.0001; del11 +Xrcc4 *vs*. flox8 +Xrcc4 p = 0.3729; del11 +Xrcc4 *vs*. flox39 +EV p = 0.4615.

To determine whether the observed phenotypes are affected by re-expression of wt*Xrcc4*, we used lentiviral transduction to express N-terminal influenza haemagglutinin (HA)-tagged wild type mouse (m)*Xrcc4* in *Xrcc4*^Δ/Δ^ 6x*Ter*-HR reporter clones #11 and #13 and in *Xrcc4*^fl/fl^ 6x*Ter*-HR reporter clones #8 and #39. Briefly, we adapted the lentiviral vector pHIV-Zsgreen [65] by replacing the *Zsgreen* cDNA with a bicistronic cDNA encoding the enzyme nourseothricin (NTC) acetyl transferase (NAT) [66] fused *via* a self-cleaving T2A peptide to the human (h)CD52 antigen (**S1A Fig**) [67]. Transient expression of the empty pHIV-NAT-CD52 vector in mouse ES cells produced strong cell surface staining of hCD52, as revealed by immunostaining using an anti hCD52-specific monoclonal antibody [68] (**S1B Fig**). Transduction of mES cells with the empty pHIV-NAT-CD52 vector, followed by selection in NTC, generated pools of transduced cells that stained strongly and specifically with anti-hCD52, whereas transduction with pHIV-NAT (i.e., lacking hCD52 expression), followed by NTC selection, generated no CD52-specific cell surface signal (**S1B Fig**). CD52 expression levels in pHIV-NAT-CD52-m*Xrcc4*-transduced, NTC-selected mES cells were lower than in control empty vector (pHIV-NAT-CD52)-transduced controls, possibly reflecting constraints imposed by *Xrcc4* expression from the multicistronic lentiviral expression cassette. Nonetheless, exogenous wt*Xrcc4* was overexpressed in comparison to endogenous *Xrcc4*, as revealed by RT-qPCR and by western blotting in lentivirally transduced *Xrcc4*^fl/fl^ cultures (**Fig 2A** and **2B**). As expected, re-expression of wt*Xrcc4* complemented the sensitivity of *Xrcc4*^Δ/Δ^ cells to the radiomimetic drug phleomycin (**Fig 2C**).

*Xrcc4*^Δ/Δ^ 6x*Ter*-HR reporter cells transduced with pHIV-NAT-CD52-*Xrcc4* and selected in NTC revealed suppression of I-SceI-induced HR to levels equivalent to that observed in isogenic *Xrcc4*^fl/fl^ 6x*Ter*-HR reporter cells (**Fig 2D**). Indeed, I-SceI-induced STGC and LTGC were each restored to wild type levels and the ratio of LTGC:Total HR reverted from ~2% to ~4% in *Xrcc4*-transduced *Xrcc4*^Δ/Δ^ cells. Parallel cultures transduced with pHIV-NAT-CD52 empty vector and selected in NTC retained the original *Xrcc4*^Δ/Δ^ phenotype. These experiments confirm that *Xrcc4* affects the balance between I-SceI-induced STGC and LTGC, suppressing STGC more strongly than LTGC. In contrast, all measures of Tus/*Ter*-induced HR were unaffected by re-expression of wt*Xrcc4* in *Xrcc4*^Δ/Δ^ cells (**Fig 2D**). To confirm these findings, and to minimize opportunities for cellular adaptation during complementation with wt*Xrcc4*, we used transient transfection to restore expression of wt*Xrcc4* in *Xrcc4*^Δ/Δ^ cells. Consistent with the above-noted findings, transient *Xrcc4* expression strongly suppressed I-SceI-induced HR in *Xrcc4*^Δ/Δ^ 6x*Ter*-HR reporter cells, but had no significant impact on Tus/*Ter*-induced STGC or LTGC in these cells (**S2 Fig**). Taken together, these experiments show that *Xrcc4* status has no impact on Tus/*Ter*-induced HR in mouse ES cells.

**Fig 3:**
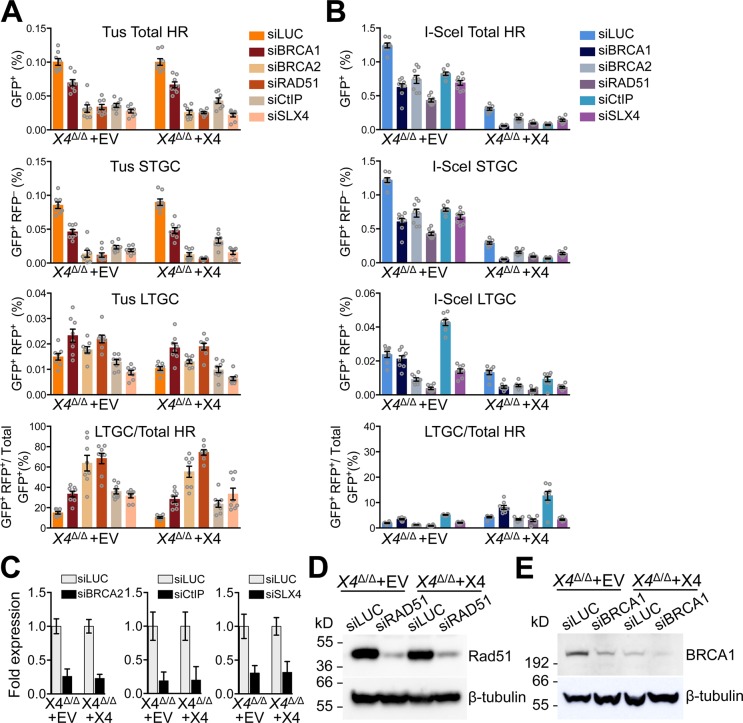
Loss of *Xrcc4* does not perturb HR regulation of Tus/*Ter*-induced STGC and LTGC. **A**, Frequencies of Tus/*Ter*-induced repair *Xrcc4*^Δ/Δ^ clone 11 6x*Ter*-HR reporter clones stably transduced with pHIV-EV (lentiviral empty vector control) or with pHIV-*mXrcc4* (HA-tagged mouse Xrcc4 lentiviral expression vector) with selection of transduced cells in 100 μg/mL NTC. Cells were transiently co-transfected with empty or 3xMyc-NLS Tus expression vectors and siRNAs as shown. Each plot represents the mean of duplicate samples from eight independent experiments (n = 8). Error bars: s.e.m. ***Xrcc4***^**Δ/Δ**^ **clone #11 pHIV-EV**: Tus-induced Total HR, t test: si*LUC* vs. si*BRCA1* p = 0.0005; si*LUC* vs. si*BRCA2* p<0.0001; si*LUC vs*. si*RAD51* p<0.0001; si*CtIP* vs. si*LUC* p<0.0001; si*SLX4* vs. si*LUC* p<0.0001. Tus-induced STGC, t test: si*LUC* vs. si*BRCA1* p<0.0001; si*LUC* vs. si*BRCA2* p<0.0001; si*LUC* vs. si*RAD51* p<0.0001; si*CtIP* vs. si*LUC* p<0.0001; si*SLX4* vs. si*LUC* p<0.0001. Tus-induced LTGC, t test: si*LUC* vs. si*BRCA1* p = 0.0153; si*LUC* vs. si*BRCA2* p = 0.1481; si*LUC* vs. si*RAD51* p = 0.0034; si*CtIP* vs. si*LUC* p = 0.2292; si*SLX4* vs. si*LUC* p = 0.0018. Tus-induced Ratio, t test: si*LUC* vs. si*BRCA1* p = 0.0001; si*LUC* vs. si*BRCA2* p = 0.0003; si*LUC* vs. si*RAD51* p<0.0001; si*CtIP* vs. si*LUC* p<0.0001; si*SLX4* vs. si*LUC* p<0.0001. ***Xrcc4***^Δ**/**Δ^ **clone #11 pHIV-*mXrcc4***: Tus-induced Total HR, t test: si*LUC* vs. si*BRCA1* p = 0.0002; si*LUC* vs. si*BRCA2* p<0.0001; si*LUC* vs. si*RAD51* p<0.0001; si*CtIP* vs. si*LUC* p<0.0001; si*SLX4* vs. si*LUC* p<0.0001. Tus-induced STGC, t test: si*LUC* vs. si*BRCA1* p<0.0001;si*LUC* vs. si*BRCA2* p<0.0001; si*LUC* vs. si*RAD51* p<0.0001; si*CtIP* vs. si*LUC* p<0.0001; si*SLX4* vs. si*LUC* p<0.0001. Tus-induced LTGC, t test: si*LUC* vs. si*BRCA1* p = 0.0023; si*LUC* vs. si*BRCA2* p = 0.0240; si*LUC* vs. si*RAD51* p = 0.0002; si*CtIP* vs. si*LUC* p = 0.7398; si*SLX4* vs. si*LUC* p = 0.0022. Tus-induced Ratio, t test: si*LUC* vs. si*BRCA1* p = 0.0004; si*LUC* vs. si*BRCA2* p<0.0001; si*LUC* vs. si*RAD51* p<0.0001; si*CtIP* vs. si*LUC* p = 0.0049; si*SLX4* vs. si*LUC* p = 0.0051. **B**, Frequencies of I-SceI-induced repair *Xrcc4*^Δ/Δ^ clone 11 6x*Ter*-HR reporter clones stably transduced with pHIV-EV (lentiviral empty vector control, “EV”) or with pHIV-*mXrcc4* (HA-tagged mouse Xrcc4 lentiviral expression vector, “X4”) with selection of transduced cells in 100 μg/ml NTC. Cells were co-transiently transfected with empty, or 3xMyc-NLS I-SceI expression vectors and siRNAs as shown. Each plot represents the mean of duplicate samples from eight independent experiments (n = 8). Error bars: s.e.m. ***Xrcc4***^Δ**/**Δ^ **clone #11 pHIV-EV**: I-SceI-induced total HR, t test: si*LUC* vs. si*BRCA1* p<0.0001; si*LUC* vs. si*BRCA2* p<0.0001; si*LUC* vs. si*RAD51* p<0.0001; si*CtIP* vs. si*LUC* p<0.0001; si*SLX4* vs. si*LUC* p<0.0001. I-SceI-induced STGC, t test: si*LUC* vs. si*BRCA1* p<0.0001; si*LUC* vs. si*BRCA2* p<0.0001; si*LUC* vs. si*RAD51* p<0.0001 si*CtIP* vs. si*LUC* p<0.0001; si*SLX4* vs. si*LUC* p<0.0001. I-SceI-induced LTGC, t test: si*LUC* vs. si*BRCA1* p = 0.3335; si*LUC* vs. si*BRCA2* p<0.0001; si*LUC* vs. si*RAD51* p<0.0001; si*CtIP* vs. si*LUC* p<0.0001; si*SLX4* vs. si*LUC* p = 0.0006. I-SceI-induced Ratio, t test: si*LUC* vs. si*BRCA1* p = 0.0001; si*LUC* vs. si*BRCA2* p = 0.0020; si*LUC* vs. si*RAD51* p<0.0001; si*CtIP* vs. si*LUC* p<0.0001; si*SLX4* vs. si*LUC* p = 0.6260. ***Xrcc4***^Δ**/**Δ^ **clone 11 pHIV-*mXrcc4***: I-SceI-induced total HR, t test: si*LUC* vs. si*BRCA1* p<0.0001; si*LUC* vs. si*BRCA2* p<0.0001; si*LUC* vs. si*RAD51* p<0.0001; si*CtIP* vs. si*LUC* p<0.0001; si*SLX4* vs. si*LUC* p<0.0001. I-SceI-induced STGC, t test: si*LUC* vs. si*BRCA1* p<0.0001; si*LUC* vs. si*BRCA2* p<0.0001; si*LUC* vs. si*RAD51* p<0.0001; si*CtIP* vs. si*LUC* p<0.0001; si*SLX4* vs. si*LUC* p<0.0001. I-SceI-induced LTGC, t test: si*LUC* vs. si*BRCA1* p = 0.0001; si*LUC* vs. si*BRCA2* p = 0.0002; si*LUC* vs. si*RAD51* p<0.0001; si*CtIP* vs. si*LUC* p = 0.0590; si*SLX4* vs. si*LUC* p = 0.0001. I-SceI-induced Ratio, t test: si*LUC* vs. si*BRCA1* p = 0.0011; si*LUC* vs. si*BRCA2* p = 0.0330; si*LUC* vs. si*RAD51* p = 0.0491; si*CtIP* vs. si*LUC* p = 0.0017; si*SLX4* vs. si*LUC* p = 0.0136. **C**, RT qPCR analysis of *BRCA1*, BRCA2, *CtIP* and *SLX4* mRNA in siRNA-treated *Xrcc4*^Δ*/*Δ^ cells stably transduced with pHIV-EV (“EV”) or pHIV-*mXrcc4* (“X4”) derived lentivirus. Data normalized to *GAPDH* and expressed as fold difference from si*LUC* sample from the same experiment (x = -2^ΔΔCt^, with ΔΔCt = [Ct _target_-Ct_Gapdh_]-[Ct_si*LUC*_-Ct_si*GAPDH*_]). Error-bars represent standard deviation of the ΔCt value (SDEV = √[SDEV_*TARGET*_^2^ + SDEV_*GAPDH*_^2^]). **D**, Western blot of RAD51 protein abundance in siRNA-treated stably transduced *Xrcc4*^Δ*/*Δ^ cells; pHIV-empty vector control (“EV”) or pHIV-*mXrcc4* (“X4”). **E**, Western blot of Brca1 protein abundance in siRNA-treated stably transduced *Xrcc4*^Δ*/*Δ^ cells; pHIV-empty vector control (“EV”) or pHIV-*mXrcc4* (“X4”).

We showed previously that STGC at Tus/*Ter*-stalled forks is controlled by the HR proteins BRCA1, CtIP, BRCA2 and Rad51 and by the structure-specific nuclease scaffold SLX4 [56, 58]. In contrast, Tus/*Ter*-induced LTGC is suppressed by BRCA1 and is independent of BRCA2 or Rad51. We found that these relationships were unaffected by *Xrcc4* status (**Fig 3A**). In the regulation of I-SceI-induced HR, we previously noted a specific role for BRCA1 and CtIP in suppressing an HR bias towards LTGC [64]. In contrast, loss of BRCA2 or Rad51 had little impact on the LTGC/Total HR ratio in response to an I-SceI-induced DSB. We observed similar effects on I-SceI-induced HR in *Xrcc4*^Δ/Δ^ 6x*Ter*-HR reporter cells (**Fig 3B**). Thus, although *Xrcc4* deletion affects the ratio of LTGC:total HR in response to I-SceI, the interactions between HR mediators in execution of HR appear to be largely unaffected by loss of C-NHEJ.

### Impact of DNA polymerase θ depletion on Tus/*Ter*- and I-SceI-induced HR

**Fig 4:**
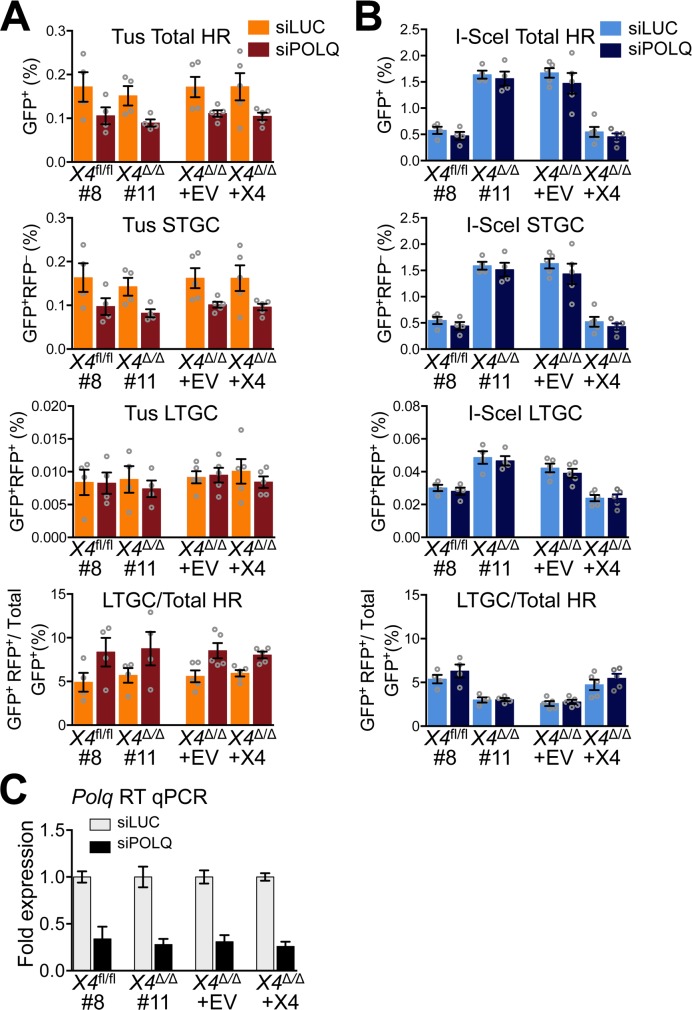
Impact of DNA polymerase θ depletion on Tus/*Ter*- and I-SceI-induced HR. **A**, Frequencies of Tus/*Ter*-induced repair in *Xrcc4*^fl/fl^ clone 8, *Xrcc4*^Δ/Δ^ clone 11, or *Xrcc4*^Δ/Δ^ clone 11 stably transduced with pHIV-EV (lentiviral empty vector control) or with pHIV-*mXrcc4* (HA-tagged mouse Xrcc4 lentiviral expression vector). Cells transiently co-transfected with empty or 3xMyc-NLS Tus expression vectors and siRNAs shown. Each plot represents the mean of duplicate samples from four (*Xrcc4*^fl/fl^ clone 8, *Xrcc4*^Δ/Δ^ clone 11) or five (*Xrcc4*^Δ/Δ^ clone 11 stably transduced cultures) independent experiments (n = 4,5). Error bars: s.e.m. Tus-induced Total HR, si*LUC* vs. si*POLQ*, t test: *Xrcc4*^fl/fl^ #8, p = 0.1534; *Xrcc4*^Δ/Δ^ #11, p = 0.0613; *Xrcc4*^Δ/Δ^ pHIV-EV, p = 0.0578; *Xrcc4*^Δ/Δ^ pHIV-*mXrcc4*, p = 0.0942. Total HR, si*LUC*, t test: *Xrcc4*^fl/fl^ #8 vs. *Xrcc4*^Δ/Δ^ #11, p = 0.6352; *Xrcc4*^Δ/Δ^ pHIV-EV, vs. pHIV-*mXrcc4*, p = 0.9841. Total HR, si*POLQ*, t test: *Xrcc4*^fl/fl^ #8 vs. *Xrcc4*^Δ/Δ^ #11, p = 0.4778; *Xrcc4*^Δ/Δ^ pHIV-EV, vs. pHIV-*mXrcc4*, p = 0.7291. Tus-induced STGC, si*LUC* vs. si*POLQ*, t test: *Xrcc4*^fl/fl^ #8, p = 0.1437; *Xrcc4*^Δ/Δ^ #11, p = 0.0510; *Xrcc4*^Δ/Δ^ pHIV-EV, p = 0.0543; *Xrcc4*^Δ/Δ^ pHIV-*mXrcc4*, p = 0.0864. STGC, si*LUC*, t test: *Xrcc4*^fl/fl^ #8 vs. *Xrcc4*^Δ/Δ^ #11, p = 0.6116; *Xrcc4*^Δ/Δ^ pHIV-EV, vs. pHIV-*mXrcc4*, p = 0.9976. STGC, si*POLQ*, t test: *Xrcc4*^fl/fl^ #8 vs. *Xrcc4*^Δ/Δ^ #11, p = 0.5041; *Xrcc4*^Δ/Δ^ pHIV-EV, vs. pHIV-*mXrcc4*, p = 0.7757. Tus-induced LTGC, si*LUC* vs. si*POLQ*, t test: *Xrcc4*^fl/fl^ #8, p = 0.9647; *Xrcc4*^Δ/Δ^ #11, p = 0.5780; *Xrcc4*^Δ/Δ^ pHIV-EV, p = 0.8273; *Xrcc4*^Δ/Δ^ pHIV-*mXrcc4*, p = 0.4575. LTGC, si*LUC*, t test: *Xrcc4*^fl/fl^ #8 vs. *Xrcc4*^Δ/Δ^ #11, p = 0.8797; *Xrcc4*^Δ/Δ^ pHIV-EV, vs. pHIV-*mXrcc4*, p = 0.6780. LTGC, si*POLQ*, t test: *Xrcc4*^fl/fl^ #8 vs. *Xrcc4*^Δ/Δ^ #11, p = 0.6918; *Xrcc4*^Δ/Δ^ pHIV-EV, vs. pHIV-*mXrcc4*, p = 0.4764. Tus-induced LTGC/(Total HR), si*LUC* vs. si*POLQ*, t test: *Xrcc4*^fl/fl^ #8, p = 0.1359; *Xrcc4*^Δ/Δ^ #11, p = 0.2154; *Xrcc4*^Δ/Δ^ pHIV-EV, p = 0.0315; *Xrcc4*^Δ/Δ^ pHIV-*mXrcc4*, p = 0.0043. LTGC/(Total HR), si*LUC*, t test: *Xrcc4*^fl/fl^ #8 vs. *Xrcc4*^Δ/Δ^ #11, p = 0.5835; *Xrcc4*^Δ/Δ^ pHIV-EV, vs. pHIV-*mXrcc4*, p = 0.6703. LTGC/(Total HR), si*POLQ*, t test: *Xrcc4*^fl/fl^ #8 vs. *Xrcc4*^Δ/Δ^ #11, p = 0.8795; *Xrcc4*^Δ/Δ^ pHIV-EV, vs. pHIV-*mXrcc4*, p = 0.4704. **B**, Frequencies of I-SceI-induced repair in *Xrcc4*^fl/fl^ clone 8, *Xrcc4*^Δ/Δ^ clone 11, or *Xrcc4*^Δ/Δ^ clone 11 stably transduced with pHIV-EV (lentiviral empty vector control) or with pHIV-*mXrcc4* (HA-tagged mouse Xrcc4 lentiviral expression vector). Cells transiently co-transfected with empty or 3xMyc-NLS I-SceI expression vectors and siRNAs shown. Each plot represents the mean of duplicate samples from four (*Xrcc4*^fl/fl^ clone 8, *Xrcc4*^Δ/Δ^ clone 11) or five (*Xrcc4*^Δ/Δ^ clone 11 stably transduced cultures) independent experiments (n = 4,5). Error bars: s.e.m. I-SceI-induced Total HR, si*LUC* vs. si*POLQ*, t test: *Xrcc4*^fl/fl^ #8, p = 0.3435; *Xrcc4*^Δ/Δ^ #11, p = 0.6415; *Xrcc4*^Δ/Δ^ pHIV-EV, p = 0.5332; *Xrcc4*^Δ/Δ^ pHIV-*mXrcc4*, p = 0.6113. Total HR, si*LUC*, t test: *Xrcc4*^fl/fl^ #8 vs. *Xrcc4*^Δ/Δ^ #11, p<0.0001; *Xrcc4*^Δ/Δ^ pHIV-EV, vs. pHIV-*mXrcc4*, p = 0.0004. Total HR, si*POLQ*, t test: *Xrcc4*^fl/fl^ #8 vs. *Xrcc4*^Δ/Δ^ #11, p = 0.0010; *Xrcc4*^Δ/Δ^ pHIV-EV, vs. pHIV-*mXrcc4*, p = 0.0178. I-SceI-induced STGC, si*LUC* vs. si*POLQ*, t test: *Xrcc4*^fl/fl^ #8, p = 0.3438; *Xrcc4*^Δ/Δ^ #11, p = 0.6464; *Xrcc4*^Δ/Δ^ pHIV-EV, p = 0.3965; *Xrcc4*^Δ/Δ^ pHIV-*mXrcc4*, p = 0.4388. STGC, si*LUC*, t test: *Xrcc4*^fl/fl^ #8 vs. *Xrcc4*^Δ/Δ^ #11, p<0.0001; *Xrcc4*^Δ/Δ^ pHIV-EV, vs. pHIV-*mXrcc4*, p<0.0001. STGC, si*POLQ*, t test: *Xrcc4*^fl/fl^ #8 vs. *Xrcc4*^Δ/Δ^ #11, p = 0.0011; *Xrcc4*^Δ/Δ^ pHIV-EV, vs. pHIV-*mXrcc4*, p = 0.0049. I-SceI-induced LTGC, si*LUC* vs. si*POLQ*, t test: *Xrcc4*^fl/fl^ #8, p = 0.5196; *Xrcc4*^Δ/Δ^ #11, p = 0.6949; *Xrcc4*^Δ/Δ^ pHIV-EV, p = 0.4229; *Xrcc4*^Δ/Δ^ pHIV-*mXrcc4*, p = 0.9733. LTGC, si*LUC*, t test: *Xrcc4*^fl/fl^ #8 vs. *Xrcc4*^Δ/Δ^ #11, p = 0.0100; *Xrcc4*^Δ/Δ^ pHIV-EV, vs. pHIV-*mXrcc4*, p = 0.0007. LTGC, si*POLQ*, t test: *Xrcc4*^fl/fl^ #8 vs. *Xrcc4*^Δ/Δ^ #11, p = 0.0028; *Xrcc4*^Δ/Δ^ pHIV-EV, vs. pHIV-*mXrcc4*, p = 0.0030. I-SceI-induced LTGC/(Total HR), si*LUC* vs. si*POLQ*, t test: *Xrcc4*^fl/fl^ #8, p = 0.3449; *Xrcc4*^Δ/Δ^ #11, p = 0.9371; *Xrcc4*^Δ/Δ^ pHIV-EV, p = 0.6062; *Xrcc4*^Δ/Δ^ pHIV-*mXrcc4*, p = 0.3769. LTGC/(Total HR), si*LUC*, t test: *Xrcc4*^fl/fl^ #8 vs. *Xrcc4*^Δ/Δ^ #11, p = 0.0090; *Xrcc4*^Δ/Δ^ pHIV-EV, vs. pHIV-*mXrcc4*, p = 0.0187. LTGC/(Total HR), si*POLQ*, t test: *Xrcc4*^fl/fl^ #8 vs. *Xrcc4*^Δ/Δ^ #11, p = 0.0181; *Xrcc4*^Δ/Δ^ pHIV-EV, vs. pHIV-*mXrcc4*, p = 0.0036. **C**, RT qPCR analysis of *POLQ* mRNA in siRNA-treated reporter cells. Data normalized to *GAPDH* and expressed as fold difference from si*LUC* sample from the same experiment (x = -2^ΔΔCt^, with ΔΔCt = [Ct_Polq_-Ct_Gapdh_]-[Ct_si*LUC*_-Ct_si*GAPDH*_]). Error-bars represent standard deviation of the ΔCt value (SDEV = √[SDEV_*TARGET*_^2^ + SDEV_*GAPDH*_^2^]).

DNA polymerase θ, encoded by the *POLQ* gene, has been implicated in an alternative end joining (A-EJ) pathway and in the prevention of genomic instability at sites of replication fork stalling [69–72]. Polθ has also been found to suppress DSB-induced HR in some cell types [73, 74]. We therefore asked whether Polθ interacts with HR in mouse ES cells, either at a Tus/*Ter* RFB or in DSB repair. Interestingly, siRNA-mediated depletion of Polθ modestly suppressed Tus/*Ter*-induced STGC in multiple clones, but in each case the effect failed reach statistical significance (**Fig 4**). Depletion of Polθ had no impact on I-SceI-induced HR either in wild type or *Xrcc4* null cells. These findings raise the possibility that Polθ supports conservative STGC at stalled forks. They also suggest that the previously reported competition between Polθ and HR in DSB repair is not a feature of mouse ES cells [73, 74].

### Impact of *Ku70* deletion on Tus/*Ter*-induced HR

**Fig 5:**
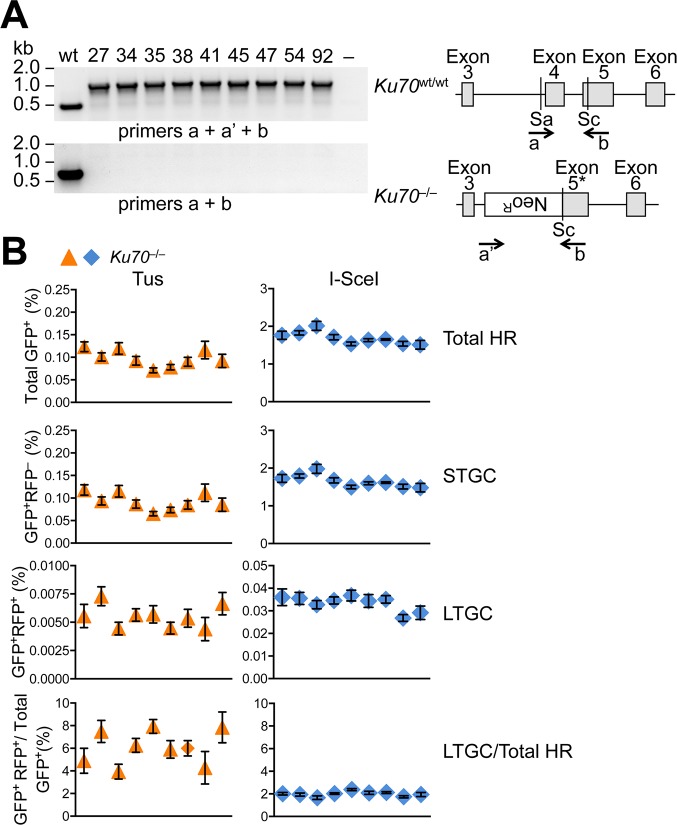
Impact of *Ku70* deletion on Tus/*Ter*-induced HR. **A**, *Ku70* mutant cell genotyping and gene structure in *Ku70*^–/–^ES cells. *Ku70* null allele lacks Exon 4 and partially Exon 5. Grey boxes: *Ku70* Exons 3–6. Location and direction of Exons 4 and 5 and *neoR* genotyping primers a, a’, and b as indicated by arrows. Sa: SacI restriction site. Sc: ScaI restriction site. Gel: PCR products for single wild type control and nine *Ku70*^–/–^ES 6x*Ter*-HR reporter clones (27, 34, 35, 38, 41, 45, 47, 54, and 92). **B**, Frequencies of Tus/*Ter*-induced and I-SceI-induced repair in nine independently derived *Ku70*^–/–^ 6x*Ter*-HR reporter clones (27, 34, 35, 38, 41, 45, 47, 54, and 92) transiently transfected with empty, 3xMyc-NLS Tus or 3xMyc-NLS I-SceI expression vectors. Each dot plot represents the mean of duplicate samples from six independent experiments (n = 6), values are corrected for transfection efficiency. Error bars: s.e.m. One-way ANOVA (Analysis of Variance) test comparing trend in HR between nine *Ku70*^–/–^clones: Tus-induced HR, total HR, p = 0.0205; STGC, p = 0.0173; LTGC, p = 0.1698; LTGC/(Total HR), p = 0.0261. I-SceI-induced HR, total HR, p = 0.0005; STGC, p = 0.0004; LTGC, p = 0.927; LTGC/(Total HR), p = 0.0081.

**Fig 6:**
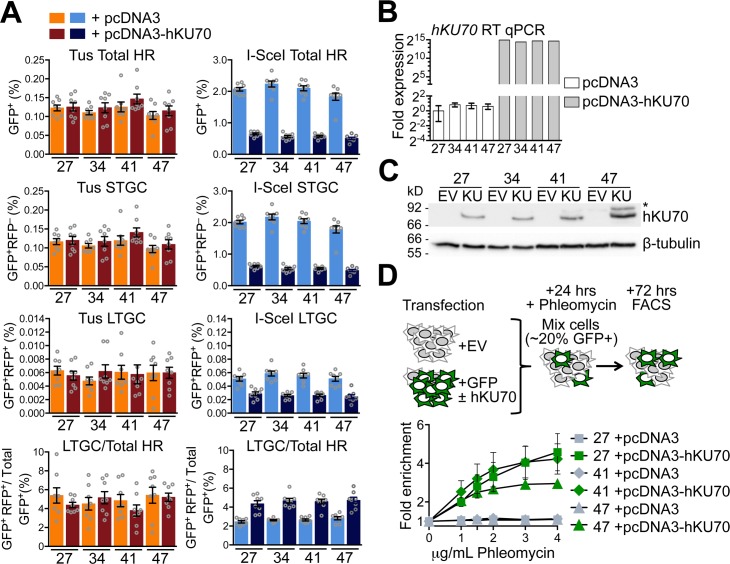
Transient *KU70* expression does not affect Tus/*Ter*-induced HR in *Ku70*^–/–^cells. **A**, Frequencies of Tus/*Ter*-induced and I-SceI-induced repair in four independently derived *Ku70*^–/–^ 6x*Ter*-HR reporter clones (clones 27, 34, 41, and 47) with and without transient expression of exogenous *hKU70*. Cells transiently co-transfected with empty pcDNA3beta or pcDNA3beta-*hKU70* expression vector and either empty, 3xMyc-NLS Tus or 3xMyc-NLS I-SceI expression vectors. Each column represents the mean of duplicate samples from eight independent experiments (n = 8), values are corrected for transfection efficiency. Error bars: s.e.m. Tus-induced total HR, t test: #27 +EV vs. +*hKU70*, p = 0.8355; #34 +EV vs. +*hKU70*, p = 0.3799; #41 +EV vs. +*hKU70*, p = 0.2710; #47 +EV vs. +*hKU70*, p = 0.4703; Tus-induced STGC, t test: #27 +EV vs. +*hKU70*, p = 0.7892; #34 +EV vs. +*hKU70*, p = 0.4223; #41 +EV vs. +*hKU70*, p = 0.2345; #47 +EV vs. +*hKU70*, p = 0.4426; Tus-induced LTGC, t test: #27 +EV vs. +*hKU70*, p = 0.4375; #34 +EV vs. +*hKU70*, p = 0.2413; #41 +EV vs. +*hKU70*, p = 0.8608; #47 +EV vs. +*hKU70*, p = 0.9872; Tus-induced LTGC/(Total HR), t test: #27 +EV vs. +*hKU70*, p = 0.2701; #34 +EV vs. +*hKU70*, p = 0.4964; #41 +EV vs. +*hKU70*, p = 0.2507; #47 +EV vs. +*hKU70*, p = 0.8222. I-SceI-induced total HR, t test: #27 +EV vs. +*hKU70*, p<0.0001; #34 +EV vs. +*hKU70*, p<0.0001; #41 +EV vs. +*hKU70*, p<0.0001; #47 +EV vs. +*hKU70*, p<0.0001; I-SceI-induced STGC, t test: #27 +EV vs. +*hKU70*, p<0.0001; #34 +EV vs. +*hKU70*, p<0.0001; #41 +EV vs. +*hKU70*, p<0.0001; #47 +EV vs. +*hKU70*, p<0.0001; I-SceI-induced LTGC, t test: #27 +EV vs. +*hKU70*, p = 0.0002; #34 +EV vs. +*hKU70*, p<0.0001; #41 +EV vs. +*hKU70*, p<0.0001; #47 +EV vs. +*hKU70*, p<0.0001; I-SceI-induced LTGC/(Total HR), t test: #27 +EV vs. +*hKU70*, p = 0.0004; #34 +EV vs. +*hKU70*, p<0.0001; #41 +EV vs. +*hKU70*, p<0.0001; #47 +EV vs. +*hKU70*, p = 0.0003. One-way ANOVA (Analysis of Variance) test comparing trend in HR: Tus-induced HR, total HR, p = 0.2388; STGC, p = 0.1923; LTGC, p = 0.9660; LTGC/(Total HR), p = 0.5923. I-SceI-induced HR, total HR, p<0.0001; STGC, p<0.0001; LTGC, p<0.0001; LTGC/(Total HR), p<0.0001. **B**, RT qPCR analysis of *hKU70* in transfected *Ku70*^–/–^clones. *hKU70* expression normalized to *GAPDH* and displayed as fold difference from *Ku70*^–/–^reporter clone 27 of the same experiment (x = -2^ΔΔCt^, with ΔΔCt = [Ct_KU70_-Ct_GAPDH_]-[Ct_*KU70*_-Ct_*GAPDH*_]). Error-bars represent standard deviation of the ΔCt value (SDEV = √[SDEV_*KU70*_^2^ + SDEV_*GAPDH*_^2^]). **C**, Western blot for abundance of hKU70 protein in *Ku70*^–/–^reporter clones transiently transfected with empty pcDNA3beta or pcDNA3beta-*hKU70*. **D**, Fold enrichment of cultures transiently expressing exogenous GFP. Results represent fold enrichment of cultures transiently co-transfected with *GFP*-expression plasmid and either pcDNA3beta or pcDNA3beta-*hKU70* expression vector over co-cultured cells transiently transfected with pcDNA3beta alone. Each plot represents the mean of triplicate samples from three independent experiments (n = 3), fold enrichment GFP+ cells normalized to 0 μg/mL phleomycin control. Error bars: s.e.m.

The binding of the Ku70/Ku80 heterodimer to DNA ends is required for engagement of C-NHEJ [75]. Ku has also been implicated in modulation of repair functions at forks stalled by the action of Topoisomerase I inhibitors, where one-ended breaks are thought to predominate [76, 77]. To determine whether Ku DNA end binding activity can influence Tus/*Ter*-induced HR independent of later steps of the C-NHEJ pathway, we targeted a single copy of the 6x*Ter*-HR reporter to the *ROSA26* locus of *Ku70*^–/–^mES cells [78]. Nine independent *ROSA26*-targeted *Ku70*^–/–^ 6x*Ter*-HR reporter clones revealed wild type levels of Tus/*Ter*-induced HR but greatly elevated levels of I-SceI-induced HR (**Fig 5**). To complement this phenotype, we co-transfected either Tus or I-SceI expression vectors with either empty vector or with a vector for expression of wt human *KU70*. Transient expression of wt*KU70* suppressed I-SceI-induced HR and complemented phleomycin sensitivity of *Ku70*^–/–^cells, as expected (**Fig 6**). In contrast, wt*KU70* expression had no impact on Tus/*Ter*-induced HR (**Fig 6**).

**Fig 7:**
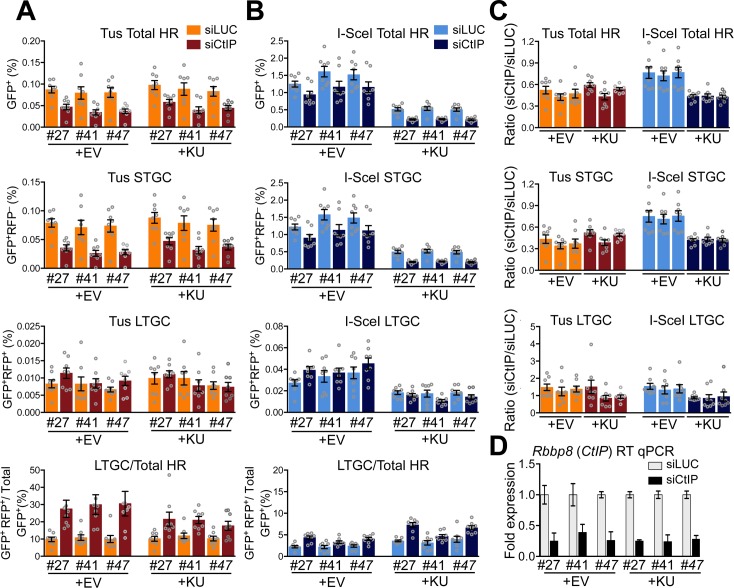
Impact of CtIP depletion on repair frequencies in the presence or absence of hKU70. **A**, Frequencies of Tus/*Ter*-induced repair in three independently derived *Ku70*^–/–^ 6x*Ter*-HR reporter clones (clones 27, 41, and 47) transiently expressing exogenous *hKU70* and transfected with siRNAs shown. Cells transiently co-transfected with empty pcDNA3beta or pcDNA3beta-*hKU70* expression vector and empty or 3xMyc-NLS Tus expression vectors treated with either si*LUC* or si*CtIP*. Each column represents the mean of duplicate samples from eight independent experiments (n = 8), values are corrected for transfection efficiency. Error bars: s.e.m. Tus-induced total HR, si*LUC* vs. si*CtIP* t test: #27 +EV, p = 0.0030; #41 +EV, p = 0.0207; #47 +EV, p = 0.0070; #27 +h*KU70*, p = 0.0047; #41 + h*KU70*, p = 0.0281; #47 + h*KU70*, p = 0.0148; Tus-induced STGC, t test: #27 +EV, p = 0.0011; #41 +EV, p = 0.0104; #47 +EV, p = 0.0070; #27 +h*KU70*, p = 0.0030; #41 + h*KU70*, p = 0.0207; #47 + h*KU70*, p = 0.0148; Tus-induced LTGC, t test: #27 +EV, p = 0.2786; #41 +EV, p = 0.5737; #47 +EV, p = 0.1304; #27 +h*KU70*, p = 0.8785; #41 + h*KU70*, p = 0.5737; #47 + h*KU70*, p = 0.5737; Tus-induced LTGC/(Total HR), t test: #27 +EV, p = 0.0002; #41 +EV, p = 0.0006; #47 +EV, p = 0.0006; #27 +h*KU70*, p = 0.0019; #41 + h*KU70*, p = 0.0030; #47 + h*KU70*, p = 0.0650; One-way ANOVA (Analysis of Variance) test comparing trend in Tus-induced Total HR: si*LUC* vs si*CtIP* all, p<0.0001; si*LUC* vs si*CtIP* +EV, p<0.0001; si*LUC* vs si*CtIP* +h*KU70*, p = 0.0002; si*LUC* all, p = 0.8904; si*CtIP* all, p = 0.1322. Tus-induced STGC, one-way ANOVA test: si*LUC* vs si*CtIP* all, p<0.0001; si*LUC* vs si*CtIP* +EV, p<0.0001; si*LUC* vs si*CtIP* +h*KU70*, p<0.0001; si*LUC* all, p = 0.9108; si*CtIP* all, p = 0.1155. Tus-induced LTGC, one-way ANOVA test: si*LUC* vs si*CtIP* all, p = 0.4334; si*LUC* vs si*CtIP* +EV, p = 0.3194; si*LUC* vs si*CtIP* +h*KU70*, p = 0.4144; si*LUC* all, p = 0.6254; si*CtIP* all, p = 0.2231. Tus-induced LTGC/(Total HR), one-way ANOVA test: si*LUC* vs si*CtIP* all, p<0.0001; si*LUC* vs si*CtIP* +EV, p = 0.0004; si*LUC* vs si*CtIP* +h*KU70*, p = 0.0012; si*LUC* all, p = 0.9449; si*CtIP* all, p = 0.2989. **B**, Frequencies of I-SceI-induced repair in three independently derived *KU70*^Δ/Δ^ 6x*Ter*-HR reporter clones. Cells transiently co-transfected with empty pcDNA3beta or pcDNA3beta-*hKU70* expression vector and empty or 3xMyc-NLS I-SceI expression vectors treated with either si*LUC* or si*CtIP*. Each column represents the mean of duplicate samples from eight independent experiments (n = 8), values are corrected for transfection efficiency. Error bars: s.e.m. I-SceI-induced total HR, si*LUC* vs. si*CtIP* t test: #27 +EV, p = 0.0379; #41 +EV, p = 0.0281; #47 +EV, p = 0.0499; #27 +h*KU70*, p = 0.0003; #41 + h*KU70*, p = 0.0019; #47 + h*KU70*, p = 0.0011; I-SceI-induced STGC, si*LUC* vs. si*CtIP* t test: #27 +EV, p = 0.0379; #41 +EV, p = 0.0281; #47 +EV, p = 0.0379; #27 +h*KU70*, p = 0.0002; #41 + h*KU70*, p = 0.0019; #47 + h*KU70*, p = 0.0011; I-SceI-induced LTGC, si*LUC* vs. si*CtIP* t test: #27 +EV, p = 0.1104; #41 +EV, p = 0.7984; #47 +EV, p = 0.3282; #27 +h*KU70*, p = 0.3282; #41 + h*KU70*, p = 0.1949; #47 + h*KU70*, p = 0.1949; I-SceI-induced LTGC/(Total HR), si*LUC* vs. si*CtIP* t test: #27 +EV, p = 0.0011; #41 +EV, p = 0.0379; #47 +EV, p = 0.0070; #27 +h*KU70*, p = 0.0006; #41 + h*KU70*, p = 0.0650; #47 + h*KU70*, p = 0.0070; I-SceI-induced Total HR, one-way ANOVA test: si*LUC* vs si*CtIP* all, p<0.0001; si*LUC* vs si*CtIP* +EV, p = 0.0139; si*LUC* vs si*CtIP* +h*KU70*, p<0.0001; si*LUC* all, p<0.0001; si*CtIP* all, p<0.0001. I-SceI-induced STGC, one-way ANOVA test: si*LUC* vs si*CtIP* all, p<0.0001; si*LUC* vs si*CtIP* +EV, p = 0.0106; si*LUC* vs si*CtIP* +h*KU70*, p<0.0001; si*LUC* all, p<0.0001; si*CtIP* all, p<0.0001. I-SceI-induced LTGC, one-way ANOVA test: si*LUC* vs si*CtIP* all, p<0.0001; si*LUC* vs si*CtIP* +EV, p = 0.1503; si*LUC* vs si*CtIP* +h*KU70*, p = 0.1010; si*LUC* all, p = 0.0010; si*CtIP* all, p<0.0001. I-SceI-induced LTGC/(Total HR), one-way ANOVA test: si*LUC* vs si*CtIP* all, p<0.0001; si*LUC* vs si*CtIP* +EV, p<0.0001; si*LUC* vs si*CtIP* +h*KU70*, p<0.0001; si*LUC* all, p = 0.0147; si*CtIP* all, p<0.0001. **C**, Observed repair frequencies for Tus or I-SceI induced HR, STGC and LTGC expressed as the ratio of si*CtIP* frequency/si*LUC* frequency for data from clones 27, 41 and 47 shown in panels **A** and **B**. One-way ANOVA test: Tus-induced total HR, p = 0.0779; Tus-induced STGC, p = 0.0564; Tus-induced LTGC, p = 0.2067. One-way ANOVA test: I-SceI-induced total HR, p<0.0001; I-SceI-induced STGC, p<0.0001; I-SceI-induced LTGC, p = 0.0832. **D**, RT qPCR analysis of *CtIP* mRNA in siRNA-transfected *Ku70*^–/–^clones. Data normalized to *GAPDH* and expressed as fold difference from si*LUC* sample from the same experiment (x = -2^ΔΔCt^, with ΔΔCt = [Ct_siCtIP_-Ct_Gapdh_]-[Ct_si*LUC*_-Ct_si*GAPDH*_]). Error-bars represent standard deviation of the ΔCt value (SDEV = √[SDEV_*CtIP*_^2^ + SDEV_*GAPDH*_^2^]).

In the processing of a conventional DSB, Ku binding to the DNA end is a barrier to DNA end resection. DNA end resection activity, initiated by CtIP and the Mre11 nuclease, can displace Ku from the DNA end, providing a mechanism by which the HR machinery can overcome the barrier formed by Ku DNA end binding [79]. To further search for evidence of Ku interaction with stalled fork HR, we determined the impact of siRNA-mediated CtIP depletion on HR in *Ku70*^–/–^cells either uncomplemented or transiently complemented with wt*KU70*. As previously reported, CtIP depletion reduced HR in response to Tus/*Ter* or to an I-SceI-mediated DSB [58], and this effect was observed in both uncomplemented and Ku70-complemented *Ku70*^–/–^cells (**Fig 7A** and **7B**). However, the proportional impact of CtIP depletion appeared less pronounced in uncomplemented I-SceI-transfected *Ku70*^–/–^cells than in the same cells complemented with wt*KU70* (**Fig 7B**). We quantified this effect by calculating, for each test group, the induced HR in cells that received siCtIP as a proportion of induced HR in cells that received the control siRNA directed to luciferase. Notably, for I-SceI-induced HR, this ratio was increased in uncomplemented *Ku70*^–/–^cells in comparison to wt*KU70*-complemented cells (**Fig 7C** and **7D**). In contrast, for Tus/*Ter*-induced HR, this ratio was unaffected by *Ku70* status. We interpret these results as follows: at a DSB, Ku binding creates a barrier to end resection and CtIP plays a significant role in displacing Ku. This Ku-displacing role of CtIP is not required in *Ku70*^–/–^cells, and the relative importance of CtIP in HR at a DSB in *Ku70*^–/–^cells is correspondingly less. In contrast, at a Tus/*Ter* RFB, CtIP plays a significant role in HR that is fully independent of *Ku70*. Taken together with the above findings with regard to *Xrcc4*, the data indicate that C-NHEJ does not compete with HR at a mammalian Tus/*Ter* RFB.

### Localized recruitment of Rad51 to a Tus/*Ter* replication fork barrier

**Fig 8:**
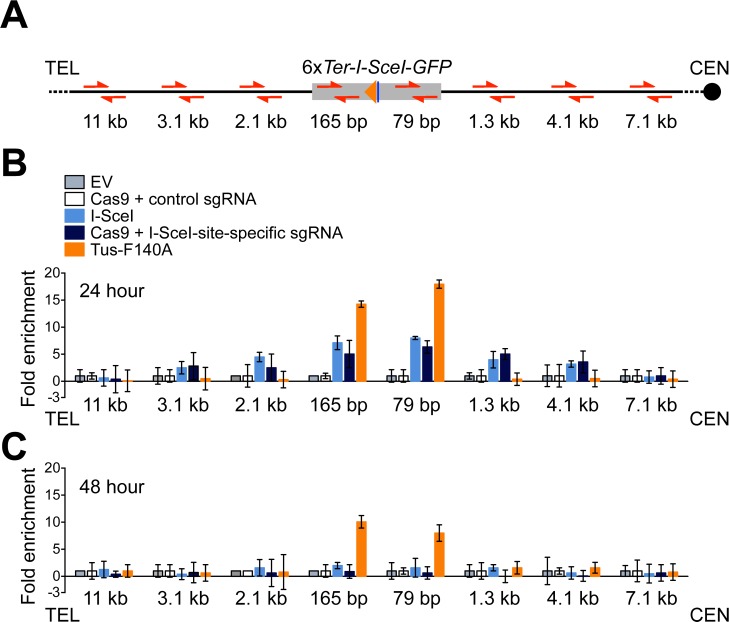
Distinct patterns of Rad51 recruitment to the Tus/*Ter* fork barrier and to a conventional DSB. A, Schematic of the 1x*GFP* 6x*Ter* reporter. Location of telomere (TEL) and centromere (CEN) shown. Red half-arrow heads: primer pairs. Grey box: mutant *GFP* allele *(*6x*Ter*-I-SceI-*GFP*). Orange triangle: 6x*Ter* element array. Navy blue line: *I-SceI* endonuclease target site and I-SceI site-specific guide RNA target site. Primer pair positions indicated as distance between the proximal end of the closer primer sequence to proximal edge of the 6x*Ter* array. B and C, Rad51 protein abundance at sites near the 6x*Ter-I-SceI-GFP* allele in response to Tus/*Ter*-induced replication fork stalling or DSB induction at 24 or 48 hours after transfection. Cells transiently transfected with empty vector (grey), pcDNA3β-myc NLS-Tus-F140A-3xHA (orange), pcDNA3β-myc NLS-I-SceI (royal blue), or co-transfected with spCas9 expression plasmid with control (white) or *I-SceI* site-specific (navy blue) guide RNA. Fold enrichment of Rad51 protein calculated as the mean 2^-ΔΔ*CT*^ from three independent experiments (n = 3) normalized against untreated controls (empty vector or guide RNA controls) and *β-Actin* control locus. Error bars indicate the standard deviation of the Δ*CT* measurement calculated as the change in Ct value obtained from the proximal-*Ter* locus and that obtained from *β-Actin* control locus.

Rad51 loading onto ssDNA is a key step in HR. In contrast to a DSB, where ssDNA is exposed following canonical DNA end resection, the stalled fork might present ssDNA for Rad51 loading through a number of different mechanisms. To determine whether Rad51 accumulates at Tus/*Ter*-stalled forks, we used chromatin-immunoprecipitation to study Rad51 accumulation at the *ROSA26* locus, in cells transfected with a DSB-inducing nuclease, Tus, or appropriate negative controls. To induce a DSB at *ROSA26*, we used either I-SceI or Cas9 targeted to the *I-SceI* target site by a sgRNA specific to the *I-SceI* site. As a negative control for I-SceI and Tus, we transfected empty expression vector. As a negative control for Cas9/*I-SceI* sgRNA, we co-transfected wtCas9 with a non-targeting sgRNA. The chromatin-immunoprecipitation method is further described in Materials and Methods. We assessed Rad51 recruitment at 24 and 48 hours following transfection, and assayed its enrichment near the 6x*Ter* array or neighboring *I-SceI* site by quantitative real-time PCR, using primers at different positions within the *ROSA26* gene (**Fig 8A**).

24 hours after transfection with either I-SceI or Cas9/*I-SceI* sgRNA, Rad51 was detected maximally at sites in close proximity to the *I-SceI* site, and this signal spread up to ~4 kb either side of the DSB (**Fig 8B**). By the 48 hour time-point, a specific DSB-induced Rad51 signal was no longer detectable (**Fig 8C**). The Rad51 response to a Tus/*Ter* RFB differed markedly. Notably, Rad51 accumulation at Tus/*Ter* was more intense than in the response to a DSB, even though Tus/*Ter* consistently induces lower HR frequencies than I-SceI in our experiments. A second striking difference was the distribution of Rad51. At the Tus/*Ter* RFB, Rad51 was strictly localized to within a few hundred base pairs of the RFB, with no spreading of the Rad51 signal detectable even 1.3 kb from the RFB. Third, the Rad51 signal remained detectable at Tus/*Ter* up to 48 hours after transfection, at a time when the DSB-induced Rad51 signal had subsided. These findings reveal that Rad51 accumulation at the Tus/*Ter* RFB is more intense, more sustained and more specifically localized than in the DSB response. Taken together, these findings suggest that the major DNA structures that bind Rad51 at a Tus/*Ter* RFB are not conventional DSBs.

## Discussion

In contrast to HR induced by a chromosomal DSB, where C-NHEJ competes to repair the two-ended break, we show here that HR induced by a Tus/*Ter* RFB in mammalian cells is unaffected by the status of the C-NHEJ genes *Xrcc4* or *Ku70*. This shows that the fundamental mechanisms of repair pathway “choice” at a stalled replication fork and a chromosomal DSB differ markedly. The simplest explanation of these findings is that HR at Tus/*Ter* does not entail formation of a two-ended DSB intermediate. We recently used High Throughput Translocation Sequencing (HTGTS) to study translocation-competent DNA lesions at Tus/*Ter* [58]. In contrast to I-SceI-induced DSBs, where two-ended breaks predominate, the major lesions detected by HTGTS at Tus/*Ter* were solitary DNA ends. However, it is possible that two-ended DSB intermediates of STGC arise at Tus/*Ter* but are not readily detected by HTGTS. Indeed, in the *X*. *laevis* model of replication-coupled ICL repair, temporally coordinated dual incisions of one sister chromatid generate a two-ended DSB intermediate. Bidirectional replication fork stalling is a critical step in this repair process, the arrival of both forks being required for replisome disassembly, asymmetrical fork reversal, nascent lagging strand resection and FANCD2/FANCI-coordinated incisions flanking the ICL [20, 23, 36].

Significant parallels exist between Tus/*Ter*-induced STGC and the above-noted model of ICL repair, especially with regard to the role of bidirectional fork arrest. We previously used Southern blotting to show that Tus/*Ter*-induced STGC products are of a fixed size, identical to products of I-SceI-induced STGC [56]. In I-SceI-induced HR, where synthesis-dependent strand annealing (SDSA) is thought to be the dominant HR pathway, the fixed size of STGC products reflects the availability of a homologous second end of the two-ended break, which supports termination of gene conversion by annealing with the displaced nascent strand [26, 27]. Indeed, if I-SceI-induced STGC is denied a homologous second end, the STGC products retrieved are of variable size, reflecting termination of gene conversion at random sites within the reporter, without the assistance of homologous pairing/annealing [64]. These aberrant STGCs are likely completed by end joining with the non-homologous second end of the DSB [80]. In the case of Tus/*Ter*-induced HR, the stereotyped structure of the STGC products implies that a homologous second DNA end was available to enable termination of STGC by annealing. This second end, we believe, must originate from the second (opposing) fork that stalls at Tus/*Ter* [56]. In summary, the mechanism of STGC at Tus/*Ter* has paradoxical properties. The structure of Tus/*Ter*-induced STGC products and its dependency on the Fanconi/BRCA/HR pathway is suggestive of SDSA of a two-ended break. However, as shown here, C-NHEJ does not compete with Tus/*Ter*-induced HR. Several possible models could reconcile these paradoxical properties.

**Fig 9:**
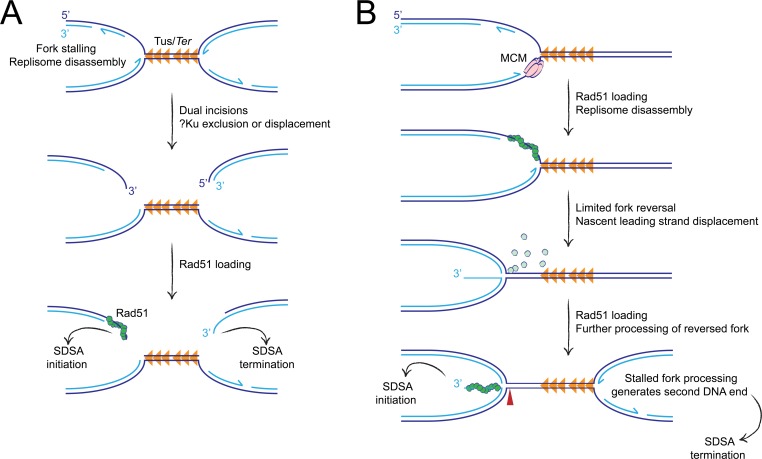
Hypothetical models of Tus/*Ter*-induced HR. **A,** Conventional DSB intermediate model. Dual incision of bidirectionally arrested forks generates DNA ends that are processed for HR. Unknown mechanisms prevent Ku access to the DNA ends at the stalled fork. Dark blue: parental strands. Light blue: nascent strands. Half arrows indicate direction of nascent strand synthesis. Orange triangles: Tus/*Ter* RFB. Green circles: Rad51 monomers. **B,** Template switch/fork reversal model. Rad51 is loaded onto exposed ssDNA lagging strand daughter strand gaps at the arrested fork. Following replisome disassembly, Rad51 mediates fork remodeling via a template switch mechanism. This process displaces the 3’ ssDNA end of the nascent leading strand, which is rapidly coated with RPA (not shown) followed by Rad51. The DNA end thus generated is incapable of binding Ku, excluding engagement of C-NHEJ. Further processing of the reversed fork may liberate the DNA end by more extensive fork reversal (not shown) and/or *via* incision of the 4-way reversed fork structure (red arrowhead). Although processing of the two opposing forks is depicted here as sequential, this model is also compatible with synchronous remodeling of both forks. Symbols as in panel A. Pale green circles, Rad51 monomers displaced from lagging strand.

In one model, the processing of the stalled fork might entail production of a conventional DSB, but the ability of Ku to access the DNA ends productively might be impaired (**Fig 9A**). Indeed, unproductive binding of Ku to presumptive solitary DNA ends at Topoisomerase I inhibitor-induced DNA lesions has been reported [76, 77]. Notably, in these studies, DNA end binding by Ku was shown to modulate repair activity and to influence the requirement for early end resection activities regulated by CtIP and Mre11. In contrast, in our experiments, deletion of *Ku70* had no impact on Tus/*Ter*-induced HR and we found no evidence of an interaction between CtIP and Ku70 in the regulation of Tus/*Ter*-induced HR. Thus, our findings do not fit readily with the idea that Ku binds unproductively to DSB intermediates during Tus/*Ter*-induced HR. In an alternative model, protein complexes at the stalled fork might deny Ku access to a conventional two-ended DSB intermediate by an as yet undefined steric exclusion mechanism. The process of V(D)J recombination in developing immune cells provides precedent for such a mechanism; the RAG protein recombination synapse both initiates incision of the recombining locus and helps to channel the DNA ends towards C-NHEJ, disfavoring engagement of alternative end joining pathways [81, 82]. However, none of our findings specifically support this model. Although inactivation of the Fanconi anemia pathway has been reported to promote C-NHEJ-mediated toxic chromosome rearrangements [59, 60], we have not yet found any genetic context in which an interaction between C-NHEJ and Tus/*Ter*-induced HR is “unmasked”.

A notable problem with the above-noted models, which invoke a conventional DSB intermediate, is their failure to account for the distinctive pattern of Rad51 accumulation we observe at Tus/*Ter*. We found that Rad51 accumulation at Tus/*Ter* is more intense, more sustained and more precisely localized than at a conventional DSB. These findings strongly suggest that the major DNA structures that recruit Rad51 to the Tus/*Ter* RFB are not conventional DSBs. We propose that Rad51 is recruited to non-DSB ssDNA structures at stalled forks and that the interaction of Rad51 with these structures accounts for the functional exclusion of C-NHEJ from stalled fork HR. A major trigger to Rad51 loading at Tus/*Ter* may be ssDNA gaps on the arrested lagging strand, present immediately adjacent to the Tus/*Ter* RFB (**Fig 9B**). Such ssDNA gaps would be present, albeit transiently, within a normally processive fork. However, fork stalling would render these same DNA structures abnormal, by virtue of their persistence. A static ssDNA signal at the site of stalling could provide a stable platform for the loading of Rad51. By this model, Rad51 might act as an “early responder” during stalled fork repair, as has been suggested previously [83]. If Rad51 deposition were a scheduled, early response to fork stalling, this might explain the intensity and localization of the Rad51 signal we observe at Tus/*Ter*.

Rad51 supports fork reversal in mammalian cells in response to a variety of DNA damaging agents [83]. Rad51-mediated template switching at the site of stalling could drive limited reversal of the collapsed fork. If initiated by Rad51-coated lagging strand gaps, this process would displace the unresected nascent leading strand as a 3’ ssDNA tail (**Fig 9B**). Rapid coating of the displaced ssDNA tail by RPA and Rad51 could render it inaccessible to binding by Ku and, hence, “invisible” to the C-NHEJ pathway. The hypothetical limited fork reversal intermediate envisioned by this model might be subject to further processing, leading to more extensive fork reversal and potentially enabling HR initiation without formation of a DSB. Alternatively, incision of the cruciate structure of the reversed fork could liberate a one-ended DSB with a long 3’ ssDNA tail formed by the displaced nascent leading strand. It is not yet clear whether Tus/*Ter*-induced HR entails the formation of such a DSB intermediate. In summary, a template switch/fork reversal model of HR initiation satisfies two of the key findings reported here: first, the intense, distinctively localized recruitment of Rad51 to the Tus/*Ter* RFB; second, the functional exclusion of C-NHEJ during Tus/*Ter*-induced HR. This hypothetical model makes a number of additional predictions, which it will be relevant to test in future studies.

An interesting feature of I-SceI-induced HR was revealed in this study. Specifically, although deletion of *Xrcc4* elevated the frequencies of both STGC and LTGC, LTGC products as a proportion of all HR products were reduced from ~4% to ~2% in *Xrcc4* null cells. *Xrcc4* deletion did not perturb the fundamental relationships of I-SceI-induced HR control reported previously for BRCA1, CtIP, BRCA2 and Rad51 [64]. This suggests that *Xrcc4* loss influences the balance between STGC and LTGC *via* an HR-independent mechanism. I-SceI-induced LTGCs, generated by the HR reporter used here, can be considered a type of gap repair [26]. Thus, I-SceI-induced LTGC might entail repair synthesis in one of two directions. The first would entail Rad51-mediated invasion of the misaligned *GFP* copy while the second would entail Rad51-mediated invasion of the correctly aligned, unbroken *I-SceI* site-containing *GFP* copy. (In the latter case, wt*GFP* would be generated by annealing at the point of SDSA termination.) In *Xrcc4*^Δ/Δ^ cells, the loss of high flux error-free religation of I-SceI-induced DSBs might increase the proportion of cells in which *I-SceI* sites on both sister chromatids are broken simultaneously. In such a circumstance, the second mechanism of LTGC noted above would be suppressed. This, in turn, could lead to the observed reduction in the proportion of I-SceI-induced HR events that resolve as LTGCs in *Xrcc4*^Δ/Δ^ cells.

## Materials and methods

### Molecular biology and siRNAs

The 6x*Ter*-HR reporters used were assembled using standard cloning methods described previously for the 6x*Ter*-HR reporter (REF). Stable *Ter*-containing plasmids were generated and manipulated in JJC33 (*Tus*^–^) mutant strains of *E*. *coli*. All primers for conventional and quantitative PCR were purchased from Life Technologies. All plasmids used for mouse embryonic stem (ES) cell transfection and 293T cell transfections were prepared by endotoxin-free maxiprep (QIAGEN Sciences, Maryland, MD). siRNA SMARTpools were purchased from GE Healthcare/Dharmacon.

### Mouse cell lines and cell culture

Conditional *Brca1* mutant mouse ES cell 1x*GFP* 6x*Ter* reporters were previously described [58]. Conditional *Xrcc4* mutant mouse ES cells (cells in which both *Xrcc4* copies contained floxed Exon3 alleles) [61] or *Ku70* mutant mouse ES cells (cells in which exon 4 and part of exon 5 is replaced with the neomycin resistance cassette [78] were thawed onto MEF feeders and subsequently maintained on gelatinized tissue culture plates in ES medium as described. 20 μg of Kpn I linearlized 6xT*er*/HR reporter *ROSA26* targeting plasmid was introduced by electroporation of 2 x 10^7^ cells. ES cells were plated onto 6-cm dishes containing Puromycin-resistant feeders and after 18 hours plates were supplemented with 4 μg/mL Puromycin for 24 hours. Individual colonies were picked for expansion between 9 and 14 days later. Multiple *ROSA26* targeted lines were identified by PCR. HR cassette *ROSA26* integration and overall structure was verified for targeted lines by Southern blotting. Multiple *Xrcc4*-deficient ES clones were generated by transient adenovirus-mediated Cre expression and excision of *Xrcc4* Exon3. *ROSA26* genotyping primers: *ROSA26-sense*-(*CAT CAA GGA AAC CCT GGA CTA CTG*); *Ter*-HR *reporter antisense*-(*cct cgg cta ggt agg gga tc*). *KU70* status was verified by PCR: *KU70 exon4 5’-sense*-(*CCA GTA AGA TCA TAA GCA GCG ATC G*); *KU70 exon5 3’-antisense*-(*CTC TTG TGA CTC ATC TTG AGC TGG*); Exon 4/5-neo-deleted allele, *KU70 3’- antisense*-(*GCC GAA TAG CCT CTC CAC CCA AGC G*). *Xrcc4* status was determined by PCR: *Xrcc4 5’-sense*-(*ttc agc taa cca gca tca ata g*); floxed allele, *Xrcc4 3’-antisense*-(*gca cct ttg cct act aag cca tct cac*); Exon 3-deleted allele, *Xrcc4 3’- antisense*-(*taa gct att act cct gca tgg agc att atc acc*). Exon3-deleted, *Xrcc4*-deficient mES cells were transduced with lentivirus expressing a single mRNA encoding nourseothricin acetyl transferase and human CD52 (the CAMPATH antigen), with or without wild type, hemagglutinin-epitope tagged mouse Xrcc4: pHIV-*NAT*-*hCD52*-EV (empty vector control) or pHIV-*NAT*-*hCD52*-*mXrcc4*. Stable cultures were selected and maintained in 100 μg/mL nourseothricin (Jenna Bioscience, AB-102L).

### Lentivirus production and target mouse cell transduction

293T cells were propagated in standard DMEM media supplemented with 10% serum, glutamine and antibiotics. For lentivirus generation, 8 x 10^6^ cells were seeded on 10 cm dishes and transfected 24 hours later with 5 μg pHIV, 4.45 μg psPAX2, and 0.55 μg pMD2G in antibiotic-free media using Lipofectamine 2000 (Invitrogen). Media was replaced 24 hours later, and supernatant harvested every 12 hours between 48 and 72 hours after transfection and stored at 4°C. Lentiviral particles were concentrated using Centricon Plus-70 filter devices (Millipore) per manufacturer’s instructions. 5 x 10^5^ target mES cells were seeded per well in 6-well plate format, allowed to proliferate for 24 hours, transduced and placed under 100 μg/mL nourseothricin selection beginning 24 hours after transduction.

### Recombination assays

1.6 x 10^5^ cells were co-transfected in suspension with 0.35 μg empty vector, pcDNA3β-myc NLS-Tus, or pcDNA3β-myc NLS-I-SceI, and 20 pmol ONTargetPlus-smartpool using Lipofectamine 2000 (Invitrogen). GFP^+^RFP^–^, GFP^+^RFP^+^ and GFP^–^RFP^+^ frequencies were scored 72 hours after transfection by flow cytometry using a Becton Dickinson 5 Laser LSRII or or Beckman Coulter CytoFlex LX in duplicate. For each duplicate sample condition, 3–6 x 10^5^ total events were scored. Repair frequencies presented are corrected for background events and for transfection efficiency (50–85%). Transfection efficiency was measured by parallel transfection with 0.05 μg wild type *GFP* expression vector, 0.30 μg control vector and 20 pmol siRNA. For transient *mXrcc4* rescue experiments, 1.6 x 10^5^ cells were co-transfected in suspension with 0.4 μg empty vector, pcDNA3β-myc NLS-Tus [56], or pcDNA3β-myc NLS-I-SceI [62], and either 0.1 μg empty vector, or pcDNA3β-HA-*Xrcc4* using Lipofectamine 2000. For transient *hKU70* rescue experiments, 1.6 x 10^5^ cells were co-transfected in suspension with 0.35 μg empty vector, pcDNA3β-myc NLS-Tus, or pcDNA3β-myc NLS-I-SceI, and either 0.15 μg empty vector, or pcDNA3β-h*KU70* using Lipofectamine 2000. For transient *hKU70* rescue experiments including siRNA treatment, 1.6 x 10^5^ cells were co-transfected in suspension with 0.35 μg empty vector, pcDNA3β-myc NLS-Tus, or pcDNA3β-myc NLS-I-SceI, and either 0.15 μg empty vector, or pcDNA3β-h*KU70* using Lipofectamine 2000 and 20 pmol siRNA.

### RT-qPCR analysis

RNA isolated from cells 48 hours after transfection was extracted using QIAGEN RNeasy Mini Kit (QIAGEN Sciences, Maryland, MD) 48 hours after transfection. All analyses of GAPDH and siRNA-targeted genes was performed using an Applied Biosystems 7300 Real time PCR System using Power SYBR Green RNA-to C_T_^TM^ 1-Step Kit (Applied Biosystems, Foster City, CA). SYBR green RT-qPCR assays were performed using gene-specific primer sequences identified using the NIH NCBI Nucleotide utility for *GAPDH*, *Slx4*, *Brca1*, *Brca2*, *CtIP*, and *Polq*. Primers for RT-PCR: *GAPDH-sense-*(*CGT CCC GTA GAC AAA ATG GT*); *GAPDH-antisense-*(*TCG TTG ATG GCA ACA ATC TC*); *Slx4-sense-*(*GTG GGA CGA CTG GAA TGA GG*); *Slx4-antisense-*(*GCA CCT TTT GGT GTC TCT GG*); *Brca1*-*sense*-(*ATG AGC TGG AGA GGA TGC TG*); *Brca1*-*antisense*-(*CTG GGC AGT TGC TGT CTT CT*); *Brca2-sense-(TCT GCC ACT GTG AAA AAT GC); Brca2-antisense*-(*TCA AGC TGG GCT GAA GAT T*); *CtIP-sense-*(*AGG AGA AGG AGG GGA CGC*); *CtIP-antisense-*(*TGA AAT ACC TCG GCG GGT G*); *Polq-sense-*(*TGC TTG GTC ACG TCT TGG AA*); *Polq-antisense-*(*CCT GAA ACA GAC TCT GGA GGT*). *mRNA* was measured in triplicates. siRNA-target gene expression level was normalized to *GAPDH* and expressed as a fold difference from si*Luciferase* control treated samples analyzed in the same experiment (x = -2^ΔΔCt^, with ΔΔCt = [Ct _target_-Ct_Gapdh_]-[Ct_si*LUCIFERASE*_-Ct_si*GAPDH*_]). Error-bars represent the standard deviation of ΔCt **(**SDEV = √[SDEV_*TARGET*_^2^ + SDEV_*GAPDH*_^2^]). We used the Roche ProbeFinder utility based on Primer 3 software (Whitehead Institute, MIT) to generate gene-specific primer sequences for mouse *Xrcc4* and human *KU70*: *Xrcc4-sense-*(*AAA TGG CTC CAC AGG AGT TG*); *Xrcc4-antisense-*(*GGT GCT CTC CTC TTT CAA GG*); *KU70-sense-*(*ACA AGT ACA GGC GGT TTG CT*); *KU70-antisense-*(*TTC AGC AGT ACC AAC GGC TT*). *Xrcc4*-specific primers mapped to exon 6 and the exon 6–7 boundary and *hKU70*-specific primers mapped to exon 7 and the exon 8, respectively. *Xrcc4* gene expression level was normalized to *GAPDH* and expressed as a fold difference from a *Xrcc4*^fl/fl^ reporter clone sample analyzed in the same experiment (x = -2^ΔΔCt^, with ΔΔCt = [Ct_Xrcc4_-Ct_Gapdh_]-[Ct_si*LUCIFERASE*_-Ct_si*GAPDH*_]). *KU70* gene expression level was normalized to *GAPDH* and expressed as a fold difference from one *Ku70*^Δ/Δ^ reporter clone sample analyzed in the same experiment (x = -2^ΔΔCt^, with ΔΔCt = [Ct_Ku70_-Ct_Gapdh_]-[Ct_si*LUCIFERASE*_-Ct_si*GAPDH*_]). Error-bars represent the standard deviation of the ΔCt value **(**SDEV = √[SDEV_*Gene*_^2^ + SDEV_*GAPDH*_^2^]).

### Western blotting

Cell lysates were prepared from cells 48 hours after transfection lysed in RIPA buffer (50mM Tris-HCl, pH 8.0, 250 mM NaCl, 0.1% sodium dodecyl sulfate, 1% NP-40 containing the protease inhibitors, PMSF, and Roche complete protease inhibitor tablet) and 10–30 μg resolved by 4–12% Bis-Tris SDS-PAGE (Invitrogen). Protein expression was analyzed by immunoblotting using the following antibodies; hRad51 (aliquot B32, 1:500), mXrcc4 (Abcam ab97351, 1:3,000), hKU70 (Thermofisher PA5-27538, 1:1000), mBrca1 (AB191042, 1:1000), HA (Abcam, ab18181, 1:500), beta-tubulin (Abcam ab6046, 1:4,000).

### Cell staining

Live cells were prepared for measurement of cell surface expression of human CD52 as previously described. Cells were trypsinized and resuspended in FACS blocking buffer (PBS containing 1% BSA, 0.1% sodium-azide, and 5% heat-inactivated goat serum). Cells were stained for CD52 in blocking buffer: primary antibody, rat anti-hCD52 mAb YTH 34.5, 1:200 (Bio-Rad AbD Serotec Inc. MCA-1642); secondary antibody, Alexa-488 AffiniPure Goat anti-Rat IgG, 1:50 (Jackson Immunoresearch, 112-545-167). Stained cells were fixed in PBS containing 0.5% BSA, 0.05% sodium-azide, 1.5% paraformaldehyde, 1% sucrose prior to flow cytometric analysis. Cell staining was measured by flow cytometry using a Becton Dickinson 5 Laser LSRII or Beckman Coulter CytoFlex LX.

### Competition assays

For *Xrcc4* mutant cell competition experiments, 1.6 x 10^5^ cells were co-transfected in suspension with 0.45 μg empty vector and either 50 ng empty vector or 50 ng *GFP*-expression plasmid using Lipofectamine 2000 (Invitrogen). For *KU70* complementation cell competition experiments, 1.6 x 10^5^ cells were co-transfected in suspension with 0.35 μg empty vector, 0.15 μg empty vector or *hKU70*-expression plasmid, and either 50 ng empty vector or 50 ng *GFP*-expression plasmid using Lipofectamine 2000 (Invitrogen). 18 hours after transfection, cells were counted, mixed 5:1, uncolored *vs*. GFP^+^ marked cells, and 5 x 10^4^ cells plated in triplicate. 6 hours after cell plating growth medium was replaced with media containing phleomycin (Sigma-aldrich, P9564). After two days incubation, GFP^+^ frequencies were scored on a Beckman Coulter CytoFlex LX. Fold enrichment of cultures transiently co-transfected with *GFP*-expression plasmid normalized to 0 μg/mL phleomycin control. Plots represent the mean of triplicate samples from three independent experiments (n = 3).

### Chromatin immunoprecipitation assays

24–48 parallel transfections of 1.6 x 10^5^ cells were performed in suspension with 0.5 μg empty vector, pcDNA3β-myc NLS-Tus-F140A-3xHA, or pcDNA3β-myc NLS-I-SceI, or co-transfected with 0.45 μg spCas9 expression plasmid with either control (*CAT CCT CGG CAC CGT CAC CC*) or I-SceI-specific (*GGA TAA CAG GGT AAT CAA GG*) guide RNAs (*in vitro* transcribed, Engen sgRNA Synthesis kit, *S*. *pyogenes*, New England Biolabs E3322S, purified using RNA Clean and Concentrator Kit, Zymo Research, R1017, and quality assessed by denaturing 10% TBE-urea acrylamide gel run) using Lipofectamine 2000 (Invitrogen). 10 million 1x*GFP* 6x*Ter* reporter cells [58] 24 or 48 hours after transfection were collected for chromatin immunoprecipitation (ChIP). Cells were fixed in serum free mES cell media containing 1% formaldehyde at room temperature, incubating for 15 min with gentle orbital shaking. Fixation was quenched by addition of glycine to 125 mM. Cells were lysed in lysis buffer (0.1% SDS, 20 mM EDTA, 50 mM Tris pH 8.1) containing protease inhibitor (PMSF supplemented with Roche protease inhibitor, Roche 13539320). All subsequent steps were performed in low DNA binding tubes (Fischer Scientific, 022431021). Chromatin shearing to 100–2000 bp was performed using Diagenode Bioruptor 300 with optional attached 4°C chiller. The predominant product size of ~500 bp as achieved by 20 sonication cycles, 15 seconds on and 30 seconds off. 100 μl lysate per ChIP reaction was precleared by addition of 10 μl Magna ChIP magnetic beads (Millipore Sigma, 16–663) in ChIP dilution buffer (1% Triton-X-100, 2mM EDTA, 150mM NaCl, 20mM Tris pH 8.1). Rad51 was immunoprecipitated by addition of 3 μg anti-Rad51 ChIP-grade antibody (Abcam, ab176458) and 12 hour incubation at 4°C on a Nutator mixer followed by addition of 10 μl Magna ChIP magnetic beads and additional 16 hour incubation at 4°C. Beads were washed six times using ice-cold ChIP RIPA buffer (50mM HEPES pH 7.6, 1mM EDTA, 7 mg/mL sodium deoxycholate, 1% NP-40). DNA was eluted in Elution buffer (1% SDS, 200mM sodium bicarbonate, 5.6 μg/mL RNAse A) and cross-links were reversed by 65°C overnight incubation. Protein was removed by proteinase K digest 30 min at 55°C. DNA purified by Qiagen PCR Purification column (Qiagen, 28106) was analyzed by qPCR using an ABI Prism 7300 sequence detection system and SYBR Green (Applied Biosystems, 4368702). Primers for qPCR: 79 bp CEN*-sense-*(*CAA CAG CCA CAA CGT CTA TAT CAT G*); 79 bp CEN*-antisense-*(*ATG TTG TGG CGG ATC TTG AAG*); 1.3 kp CEN*-sense-*(*CAC CAC AAA TCG AGG CTG TA*); 1.3 kp CEN*-antisense-*(*GGA TCA AGG CAA AGG ATC AA*); 4.1 kp CEN*-sense-*(*TCC GGT GAA TAG GCA GAG TT*); 4.1 kp CEN*-antisense-*(*CAG GGA AAC CCA AAG AAG TG*); 7.1 kp CEN*-sense-*(*TGC AAA AAC CAT CCA AAC AA*); 7.1 kp CEN*-antisense-*(*GTG GAG GCT AGA AGC TGG TG*); 165 bp TEL*-sense-*(*TGG TGA GCA AGG GCG AGG AGC*); 165 bp TEL*-antisense-*(*TCG TGC TGC TTC ATG TGG TCG*); 2.1 kp TEL*-sense-*(*GGG AGG CTA ACT GAA ACA CG*); 165 bp TEL*-antisense-*(*GGT GGG GTA TCG ACA GAG TG*); 3.1 kp TEL*-sense-*(*GCA CGT TTC CGA CTT GAG TT*); 165 bp TEL*-antisense-*(*TCA GAG CGA CTT TGG GAG AG*); 11 kp TEL*-sense-*(*CAG GAA TTC TTT CCC CAC AA*); 165 bp TEL*-antisense-*(*TGC CAG GTC TCT AGG GCT TA*). Data are presented as the mean calculated from three independent experiments (n = 3) normalized against untreated controls (empty vector or guide RNA controls) and control locus (beta-actin) using the 2^-**ΔΔ*CT***^ method as described previously [84].

### Statistical methods

Data presented represents the arithmetic mean and error bars represent the standard error of the mean (s.e.m.) of between three (n = 3) and nine (n = 9) independent experiments (n values given in figure legends). Figure legends specify the number of replicates within each independent experiment (performed in duplicate) and the number of independent experiments (n) that were performed to generate the data presented. The arithmetic mean of samples collected for groups of independent experiments for repair frequency statistical analysis, was calculated and data points for each independent experiment used to calculate the mean and standard error of the mean (s.e.m.), calculated as standard deviation/√n, (n indicates the number of independent experiments). Differences between sample pairs repair frequencies were analyzed by Student’s two-tailed unpaired *t*-test, assuming unequal variance. One-way ANOVA statistical analysis of greater than three samples was performed when indicated. P-values are indicated in the figure legends. All statistics were performed using GraphPad Prism v6.0d software.

## Supporting information

S1 Fig A mammalian nourseothricin multicistronic lentiviral selection system. **A**, Map of pHIV-NAT-hCD52 indicating key elements and location of *mXrcc4* cDNA insert: EF1α: elongation factor alpha promoter; IRES: internal ribosomal entry signal; NAT: nourseothricin (NTC) acetyl transferase resistance gene: T2A, Thosea asigna viral self-cleaving peptide; CD52: human CAMPATH antigen cDNA. **B**, Primary FACS histogram data for mES reporter cells expressing human CD52. Left panel: *Xrcc4*^Δ*/*Δ^ reporter cells transiently transfected with pHIV-NAT-hCD52 vector, stained with either no primary antibody (grey) or anti-hCD52 mAb YTH 34.5 (green). Right panel: hCD52 cell surface expression in *Xrcc4*^Δ*/*Δ^ reporter cells following lentiviral transduction and selection in 100 μg/ml NTC. Gray histogram: pHIV-NAT-transduced cells. Green histogram: pHIV-NAT-hCD52-transduced cells. Inset numbers indicate median CD52 staining intensity.
(PDF)

S2 Fig Transient *Xrcc4* expression does not affect Tus/*Ter*-induced HR in *Xrcc4*^Δ/Δ^ cells. **A**, Frequencies of Tus/*Ter*-induced and I-SceI-induced repair of *Xrcc4*^fl/fl^ clone #8 or *Xrcc4*^Δ/Δ^ clone #11 6x*Ter*-HR reporter cells transiently expressing exogenous *Xrcc4*. Cells were transiently co-transfected with empty pcDNA3beta or pcDNA3beta-HA-*mXrcc4* expression vector and empty, 3xMyc-NLS Tus or 3xMyc-NLS I-SceI expression vectors. Each plot represents the mean of duplicate samples from nine independent experiments (n = 9). Error bars: s.e.m. Tus-induced Total HR, t-test: flox8 +Xrcc4 *vs*. flox8 +EV p = 0.0048; del11 +Xrcc4 *vs*. del11 +EV p = 0.7251; del11 +EV *vs*. flox8 +EV p = 0.0002; del11 +Xrcc4 *vs*. flox8 +Xrcc4 p = 0.4659; del11 +Xrcc4 *vs*. flox8 +EV p = 0.0005. Tus-induced STGC, t-test: flox8 +Xrcc4 *vs*. flox8 +EV p = 0.0035; del11 +Xrcc4 *vs*. del11 +EV p = 0.7076; del11 +EV *vs*. flox8 +EV p = 0.0003; del11 +Xrcc4 *vs*. flox8 +Xrcc4 p = 0.4650; del11 +Xrcc4 *vs*. flox8 +EV p = 0.0005. Tus-induced LTGC, t-test: flox8 +Xrcc4 *vs*. flox8 +EV p = 0.5738; del11 +Xrcc4 *vs*. del11 +EV p = 0.8987; del11 +EV *vs*. flox8 +EV p = 0.3906; del11 +Xrcc4 *vs*. flox8 +Xrcc4 p = 0.8369; del11 +Xrcc4 *vs*. flox8 +EV p = 0.5332. Tus-induced Ratio, t-test: flox8 +Xrcc4 *vs*. flox8 +EV p = 0.0513; del11 +Xrcc4 *vs*. del11 +EV p = 0.7973; del11 +EV *vs*. flox8 +EV p = 0.2700; del11 +Xrcc4 *vs*. flox8 +Xrcc4 p = 0.7369; del11 +Xrcc4 *vs*. flox8 +EV p = 0.1734. I-SceI-induced Total HR, t-test: flox8 +Xrcc4 *vs*. flox8 +EV p = 0.0398; del11 +Xrcc4 *vs*. del11 +EV p<0.0001; del11 +EV *vs*. flox8 +EV p<0.0001; del11 +Xrcc4 *vs*. flox8 +Xrcc4 p = 0.5330; del11 +Xrcc4 *vs*. flox8 +EV p = 0.0034. I-SceI-induced STGC, t-test: flox8 +Xrcc4 *vs*. flox8 +EV p = 0.0391; del11 +Xrcc4 *vs*. del11 +EV p<0.0001; del11 +EV *vs*. flox8 +EV p<0.0001; del11 +Xrcc4 *vs*. flox8 +Xrcc4 p = 0.5491; del11 +Xrcc4 *vs*. flox8 +EV p = 0.0039. I-SceI-induced LTGC, t-test: flox8 +Xrcc4 *vs*. flox8 +EV p = 0.4390; del11 +Xrcc4 *vs*. del11 +EV p<0.0001; del11 +EV *vs*. flox8 +EV p = 0.0002; del11 +Xrcc4 *vs*. flox8 +Xrcc4 p = 0.6173; del11 +Xrcc4 *vs*. flox8 +EV p = 0.2036. I-SceI-induced Ratio, t-test: flox8 +Xrcc4 *vs*. flox8 +EV p = 0.9640; del11 +Xrcc4 *vs*. del11 +EV p<0.0001; del11 +EV *vs*. flox8 +EV p = 0.0012; del11 +Xrcc4 *vs*. flox8 +Xrcc4 p = 0.8666; del11 +Xrcc4 *vs*. flox8 +EV p = 0.9242. **B**, RT qPCR analysis of *Xrcc4* in transfected *Xrcc4*^fl/fl^ or *Xrcc4*^Δ*/*Δ^ clones. *Xrcc4* expression normalized to *GAPDH* and displayed as fold difference from *Xrcc4*^fl/fl^ parental reporter clone 8 of the same experiment (x = -2^ΔΔCt^, with ΔΔCt = [Ct_Xrcc4_-Ct_Gapdh_]-[Ct_*Xrcc4*_-Ct_*GAPDH*_]). Error-bars represent standard deviation of the ΔCt value (SDEV = √[SDEV_*Xrcc4*_^2^ + SDEV_*GAPDH*_^2^]). **C**, Western blot for abundance of HA-tagged mXrcc4 protein in *Xrcc4*^fl/fl^ clone 8 or *Xrcc4*^Δ/Δ^ clone 11 6x*Ter*-HR reporter cells transiently transfected with empty pcDNA3beta or pcDNA3beta-*mXrcc4*.
(PDF)
