# Supplementary figures and images for: Rad51 recruitment and exclusion of non-homologous end joining during homologous recombination at a Tus/Ter mammalian replication fork barrier
Source: PLoS Genet. 2018 Jul 19;14(7):e1007486. doi: 10.1371/journal.pgen.1007486 (PMC6067765; doi:10.1371/journal.pgen.1007486)

**A**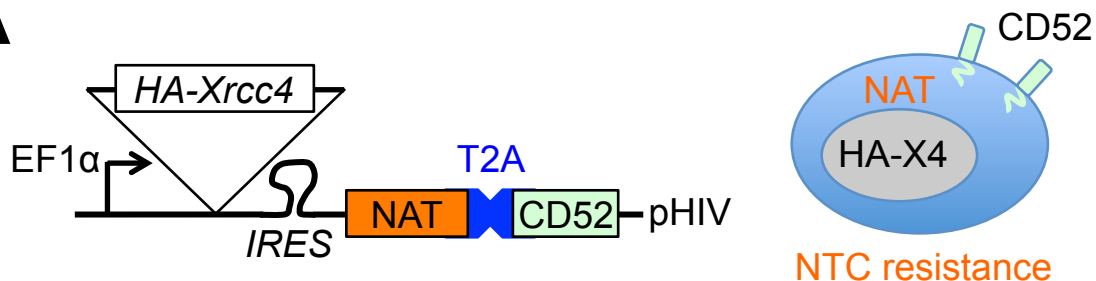**B**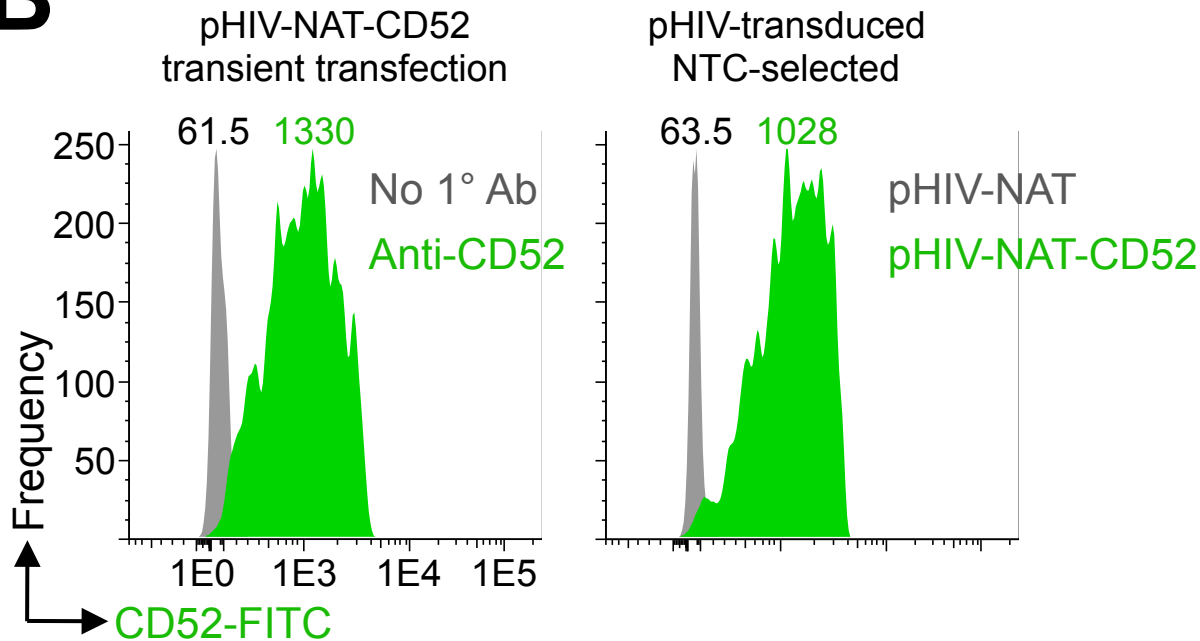

Supplement: S1 Fig — A, Map of pHIV-NAT-hCD52 indicating key elements and location of mXrcc4 cDNA insert: EF1α: elongation factor alpha promoter; IRES: internal ribosomal entry signal; NAT: nourseothricin (NTC) acetyl transferase resistance gene: T2A, Thosea asigna viral self-cleaving peptide; CD52: human CAMPATH antigen cDNA. B, Primary FACS histogram data for mES reporter cells expressing human CD52. Left panel: Xrcc4Δ/Δ reporter cells transiently transfected with pHIV-NAT-hCD52 vector, stained with either no primary antibody (grey) or anti-hCD52 mAb YTH 34.5 (green). Right panel: hCD52 cell surface expression in Xrcc4Δ/Δ reporter cells following lentiviral transduction and selection in 100 μg/ml NTC. Gray histogram: pHIV-NAT-transduced cells. Green histogram: pHIV-NAT-hCD52-transduced cells. Inset numbers indicate median CD52 staining intensity. (PDF) [file pgen.1007486.s001.pdf]

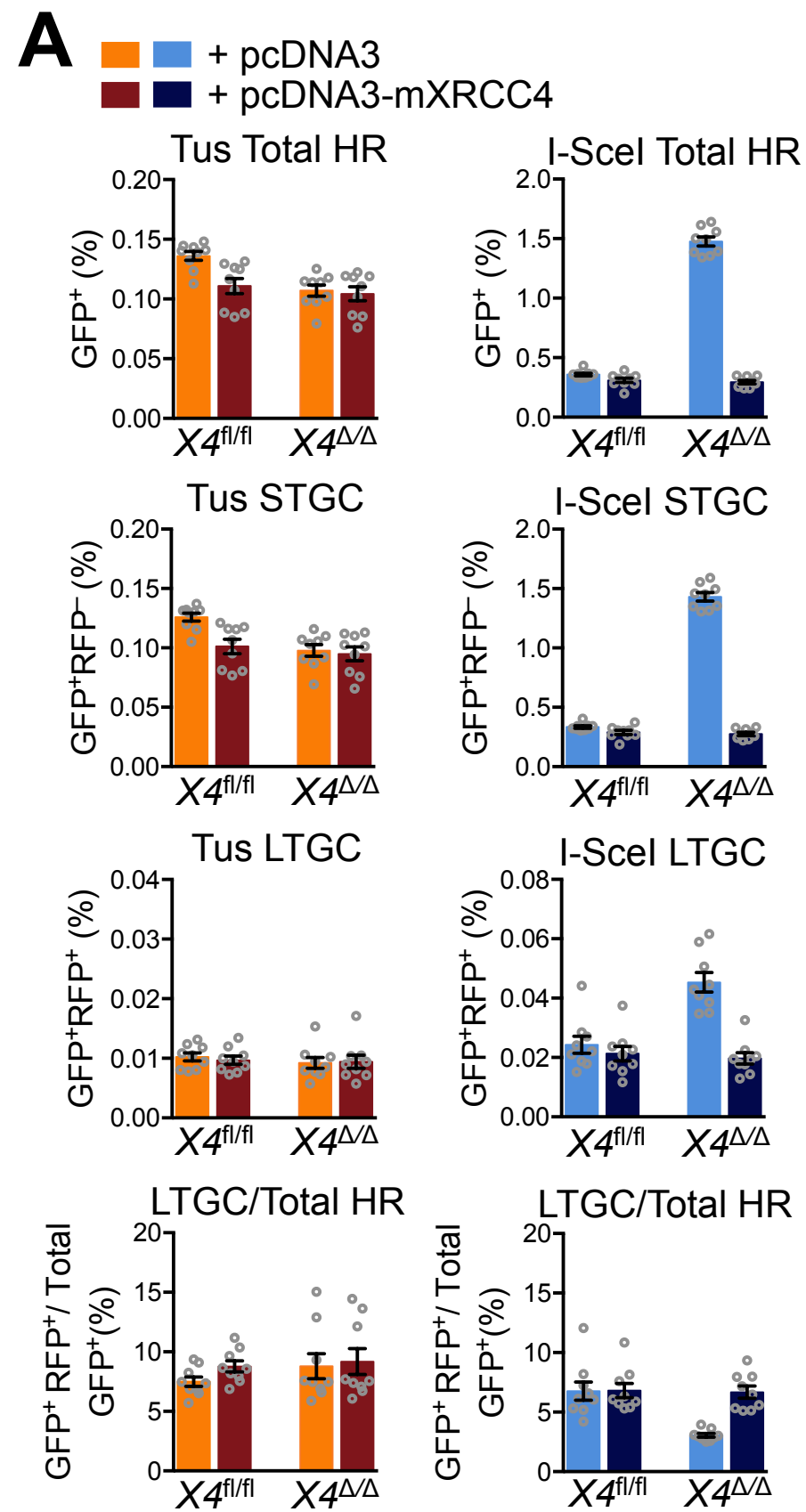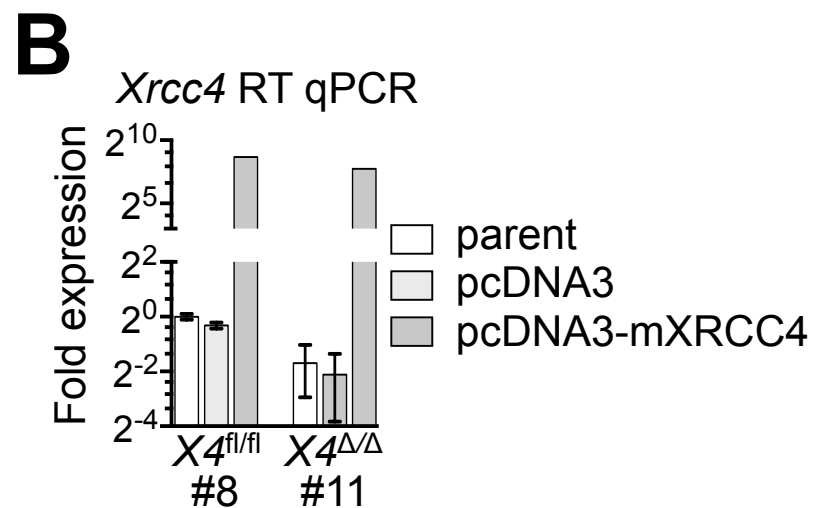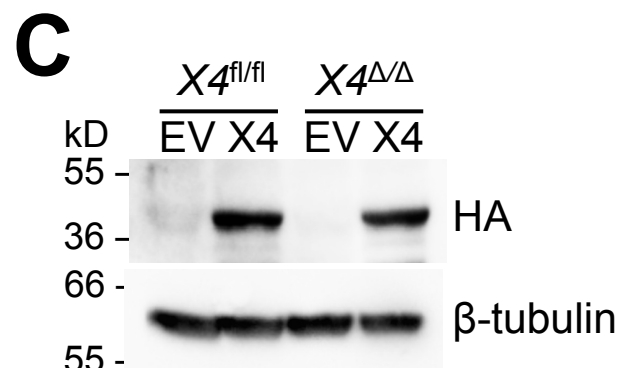

Supplement: S2 Fig — A, Frequencies of Tus/Ter-induced and I-SceI-induced repair of Xrcc4fl/fl clone #8 or Xrcc4Δ/Δ clone #11 6xTer-HR reporter cells transiently expressing exogenous Xrcc4. Cells were transiently co-transfected with empty pcDNA3beta or pcDNA3beta-HA-mXrcc4 expression vector and empty, 3xMyc-NLS Tus or 3xMyc-NLS I-SceI expression vectors. Each plot represents the mean of duplicate samples from nine independent experiments (n = 9). Error bars: s.e.m. Tus-induced Total HR, t-test: flox8 +Xrcc4 vs. flox8 +EV p = 0.0048; del11 +Xrcc4 vs. del11 +EV p = 0.7251; del11 +EV vs. flox8 +EV p = 0.0002; del11 +Xrcc4 vs. flox8 +Xrcc4 p = 0.4659; del11 +Xrcc4 vs. flox8 +EV p = 0.0005. Tus-induced STGC, t-test: flox8 +Xrcc4 vs. flox8 +EV p = 0.0035; del11 +Xrcc4 vs. del11 +EV p = 0.7076; del11 +EV vs. flox8 +EV p = 0.0003; del11 +Xrcc4 vs. flox8 +Xrcc4 p = 0.4650; del11 +Xrcc4 vs. flox8 +EV p = 0.0005. Tus-induced LTGC, t-test: flox8 +Xrcc4 vs. flox8 +EV p = 0.5738; del11 +Xrcc4 vs. del11 +EV p = 0.8987; del11 +EV vs. flox8 +EV p = 0.3906; del11 +Xrcc4 vs. flox8 +Xrcc4 p = 0.8369; del11 +Xrcc4 vs. flox8 +EV p = 0.5332. Tus-induced Ratio, t-test: flox8 +Xrcc4 vs. flox8 +EV p = 0.0513; del11 +Xrcc4 vs. del11 +EV p = 0.7973; del11 +EV vs. flox8 +EV p = 0.2700; del11 +Xrcc4 vs. flox8 +Xrcc4 p = 0.7369; del11 +Xrcc4 vs. flox8 +EV p = 0.1734. I-SceI-induced Total HR, t-test: flox8 +Xrcc4 vs. flox8 +EV p = 0.0398; del11 +Xrcc4 vs. del11 +EV p<0.0001; del11 +EV vs. flox8 +EV p<0.0001; del11 +Xrcc4 vs. flox8 +Xrcc4 p = 0.5330; del11 +Xrcc4 vs. flox8 +EV p = 0.0034. I-SceI-induced STGC, t-test: flox8 +Xrcc4 vs. flox8 +EV p = 0.0391; del11 +Xrcc4 vs. del11 +EV p<0.0001; del11 +EV vs. flox8 +EV p<0.0001; del11 +Xrcc4 vs. flox8 +Xrcc4 p = 0.5491; del11 +Xrcc4 vs. flox8 +EV p = 0.0039. I-SceI-induced LTGC, t-test: flox8 +Xrcc4 vs. flox8 +EV p = 0.4390; del11 +Xrcc4 vs. del11 +EV p<0.0001; del11 +EV vs. flox8 +EV p = 0.0002; del11 +Xrcc4 vs. flox8 +Xrcc4 p = 0.6173; del11 +Xrcc4 vs. flox8 +EV p = 0.20 [file pgen.1007486.s002.pdf]
